# Non-neoplastic hepatopancreatobiliary lesions simulating malignancy: can we differentiate?

**DOI:** 10.1186/s13244-019-0813-8

**Published:** 2020-02-10

**Authors:** Ali Devrim Karaosmanoglu, Aycan Uysal, Musturay Karcaaltincaba, Deniz Akata, Mustafa Nasuh Ozmen, Jessica Kraeft, Peter F. Hahn

**Affiliations:** 1 Department of Radiology, Hacettepe University School of Medicine, 06100 Ankara, Turkey; 2 Department of Radiology, Gulhane Training and Research Hospital, 06010 Ankara, Turkey; 3 Department of Radiology, University of Colorado School of Medicine, Aurora, CO 80045 USA; 4 Department of Radiology, Massachusetts General Hospital, Harvard Medical School, Boston, MA 02114 USA

**Keywords:** Hepatobiliary, Pancreas, Neoplasm, Mimicker, Radiology

## Abstract

Despite the success of cross-sectional imaging in evaluating hepatopancreatobiliary system malignancies, several non-malignant disease processes may closely mimic malignancy. Differentiating these benign diseases from malignancy may be difficult, or even impossible, even in the hands of experienced imagers. In this manuscript, we present benign mimics involving the hepatopancreatobiliary system and try to increase awareness of these potential pitfalls.

## Key points

- Several infectious and inflammatory conditions may mimic neoplastic processes on imaging studies and differential diagnosis may be difficult.
- Clinical history and patient demographics are critical for correct diagnosis in addition to imaging findings.
- Image-guided biopsy may be used for definitive differential diagnosis in selected patients.

## Introduction

Despite the success of cross-sectional imaging in evaluating hepatopancreatobiliary system malignancies, several non-malignant disease processes may closely mimic malignancy [1, 2]. Differentiating these benign diseases from malignancy may be difficult, or even impossible, even in the hands of experienced imagers. In this manuscript, we present benign mimics involving the hepatopancreatobiliary system and try to increase awareness of these potential pitfalls.

### Liver

Liver is probably the most commonly affected organ in the abdomen in patients with malignancy. Secondary malignancies, in the form of metastases from different sources, far exceed the primary malignant diseases of the liver. The imaging diagnosis of primary hepatic tumors including hepatocellular carcinoma (HCC) and cholangiocellular carcinomas (CCC) is usually straightforward. Metastatic involvement of the liver is common and diagnosis is generally not problematic in patients with known primary malignancies outside the liver. The early detection of metastatic liver disease is important; as it is generally a sign of advanced disease and the patient’s treatment and prognostic expectations should be adjusted accordingly in the light of this finding. Both infectious and non-infectious tumor-like conditions mimic primary and secondary liver neoplasms. We will discuss the infectious mimics first, followed by non-infectious disease processes which may simulate a liver malignancy.

### Infectious mimics

The liver is frequently affected in the course of various viral, bacterial, fungal, and parasitic infections. There are several predisposing local and systemic factors which may render liver more prone to infections, including, but not limited to, immunosuppression such as related to human immunodeficiency virus (HIV), several drugs, and surgery. The differential diagnosis may be difficult and a detailed patient history is critical for correct diagnosis.

## *Candida albicans* infection

Fungal liver infections are most common in patients with compromised immune system or in cases of hematologic malignancies. It is a manifestation of systemic fungal infection [3]. Leukemia is the most commonly encountered predisposing factor for hepatic infection from *Candida albicans* [3].

Ultrasound (US), computed tomography (CT), and magnetic resonance imaging (MRI) may all be used for diagnosis. On US, the echogenicity of the parenchymal candida nodules vary significantly depending on the stage of the disease [4]. The most frequent patterns is detection of multiple hypoechoic subcentimeter lesions which may closely mimic lymphomatous or leukemic involvement as well as metastases from solid organ cancers [5].

**Fig. 1:**
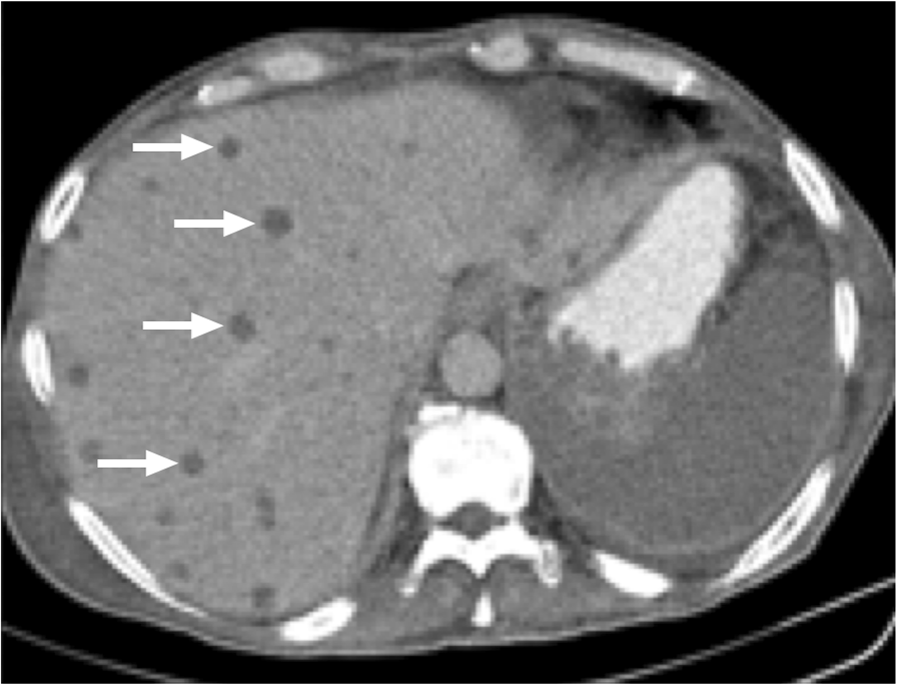
A 61-year-old male with recently diagnosed AML undergoing chemotherapy now presenting with neutropenic fever. Axial plane postcontrast CT image demonstrates multiple well-defined hypodense liver lesions (arrows). Clinical and laboratory findings were consistent with candida infection. The findings almost completely resolved after antifungal treatment (not shown)

CT is a very commonly used modality for diagnosis. The infectious parenchymal foci mostly appear as round low attenuation lesions with distinct margins (Fig. 1). The size generally ranges from 2 to 20 mm. Detection of a hyperattenuating rim surrounding a hypoattenuating center (“bull's eye” appearance) in the arterial phase of the scan appears to be the most sensitive finding. In the portal-venous phase, lesions are often seen as subcentimeter hypoattenuating lesions [5–7].

On MRI, the nodules appear hypointense on pre-contrast T1-weighted (T1W) images with marked hyperintensity on corresponding T2-weighted (T2W) images [8, 9]. Enhancement may be seen after contrast injection [9]. The signal intensities may evolve during the course of the disease. The T2 signal gradually fades away with the response to treatment [10]. MRI was reported to be superior to CT for detection of these fungal foci [8, 10].

Differential diagnosis may be difficult and these lesions may easily be confused with metastatic disease. Clinical context and patient history are key factors for correct diagnosis. In patients who are undergoing an intensive chemotherapy for hematological malignancies, the acute emergence of fever, elevated serum CRP, and abnormal liver function tests are highly suggestive for hepatic candidiasis. Follow-up imaging after treatment may be helpful for confirming the diagnosis in some patients [11]. Sometimes, lesions paradoxically become more evident as the white count recovers and patient’s condition improves. Biopsy is typically not indicated for diagnosis in the setting of suggestive clinical history [1]. Despite all efforts, conclusive diagnosis may be impossible based on imaging findings and a percutaneous biopsy procedure may be needed for final diagnosis. Fungal micro-abscesses commonly involve the spleen as well. The spleen should be carefully scrutinized for coincident infection.

### Hepatic tuberculosis

Tuberculosis (TB) is globally among the most common infectious diseases, with a variable distribution around the world. Liver involvement may be classified as either miliary form, where liver is involved in the course of systemic disease or as mass-forming localized form. The localized mass-forming (macronodular, > 2 cm) hepatic TB lesions are typically seen as focal parenchymal lesions. Hepatic tuberculomas and tuberculous abscesses may be detected in this form [12–14].

As isolation of the slow-growing TB bacilli may be extremely difficult for definite diagnosis, imaging plays a crucial role in the diagnosis. Miliary lesions are typically observed as multiple subcentimeter lesions (generally less than 2 cm in size). These may be randomly distributed throughout the liver parenchyma but sometimes associated in clusters. These lesions may appear as solid or cystic, with minimal peripheral contrast enhancement. It may sometimes be difficult to differentiate them from metastases or lymphoma involvement [14].

**Fig. 2:**
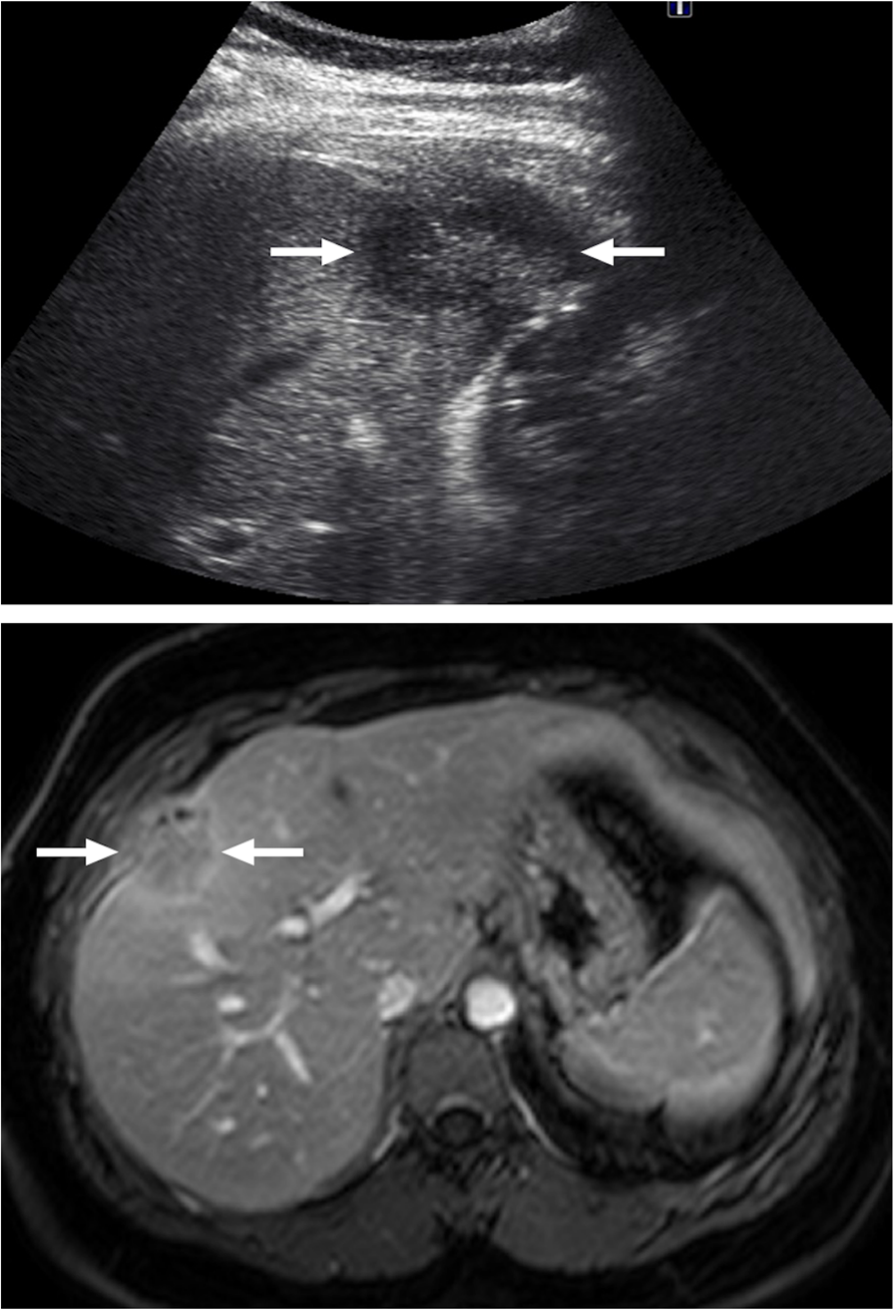
A 37-year-old female with no known systemic disease presented with right upper quadrant pain. **a** Abdominal ultrasound demonstrates hypoechoic solid mass lesion with slightly increased central echogenicity (arrows). **b** Axial plane postcontrast T1W image demonstrates peripherally enhancing infiltrative sub-capsular mass lesion (arrows) extending into perihepatic area and infiltrating abdominal wall fat. Also note associated peritoneal thickening. Percutaneous US-guided biopsy revealed granulomatous inflammation consistent with tuberculoma

Tuberculomas are usually detected as round lesions and may manifest as solitary or multiple lesions of varying sizes. This form is rare as compared to the miliary form and may closely mimic metastatic disease or a primary liver tumor. Tuberculomas are generally larger than 2 cm in size and are also referred to as macronodular or pseudotumoral TB [14]. Tuberculomas may be seen as hypoechoic solid masses on US; however, hyperechogenicity may also be occasionally seen [15–17]. On CT, the imaging findings are generally not conclusive and they are mostly observed as hypoattenuating lesions with ill-defined borders with surrounding edema. MRI findings are also not very specific and they are usually hypointense on T1W images and hypo, iso, or hyperintense on T2W images with a peripheral hypointense rim [18]. Postcontrast images typically demonstrate peripheral rim-shaped enhancement with occasional heterogenous septal enhancement (Fig. 2) [13, 14]. The peripheral enhancement pattern may be useful for differentiating tuberculomas from neoplastic disease such as HCC and hypervascular metastases [13]. Cystic metastases from the ovaries and gastrointestinal system may look similar but detection of the perilesional edema, which is more conspicuous on T2W images, is more characteristic for a TB or pyogenic abscess over a cystic metastasis.

Hepatic TB should be considered in the differential diagnosis in patients with suggestive clinical findings such as documented TB elsewhere in the body or a history of prior TB. The demographics of the patient as well as the country of origin (in migrant and refugee patients) may also be other helpful clinical clues. However, percutaneous biopsy should be needed in certain clinical circumstances where noninvasive definitive diagnosis cannot be done.

### Hydatid disease

**Fig. 3:**
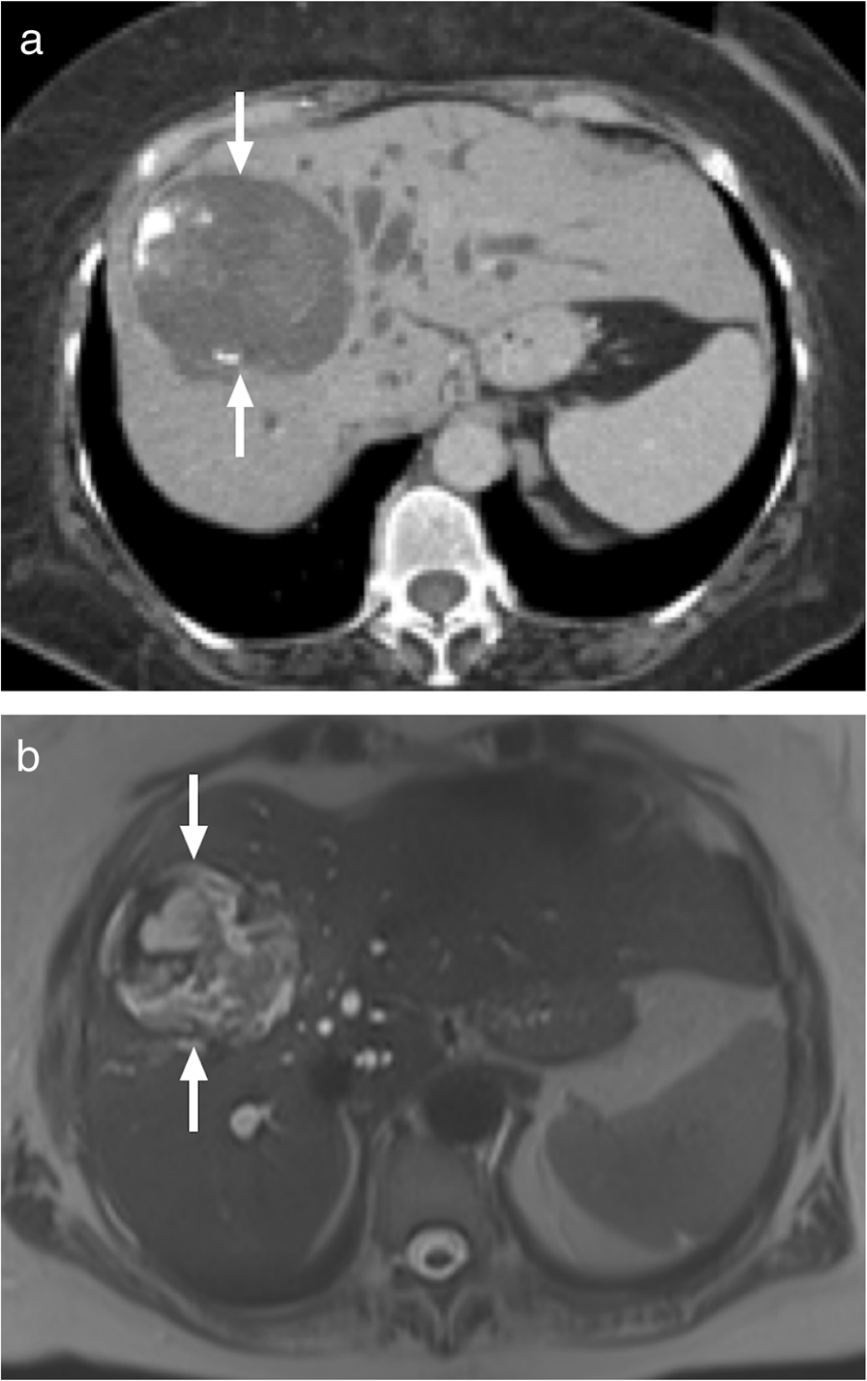
A 77-year-old male farmer with no significant medical history presented with recently onset jaundice and right upper quadrant pain. **a** Axial plane postcontrast abdominal CT demonstrates a complex looking mass with heterogenous central part and peripheral calcifications (arrows). Also note dilatation of the intrahepatic bile ducts due to the mass effect of the lesion. **b** Axial plane T2W MR image demonstrates the cystic nature of the mass (arrows) with internal septations which may suggest detached eccinochocal membrane. There was no evidence of internal contrast enhancement on postcontrast dynamic T1W images (not shown). Percutaneous drainage confirmed hydatid cyst

Hydatid disease occurs in humans incidentally infected with *Echinococcus granulosus* and *Echinococcus multilocularis*. Right lobe is the most frequently involved part of the liver in patients infected with the cystic hydatid disease caused by *E*. *granulosus*. The imaging findings are highly variable depending on the stage of the disease. US primarily shows anechoic or multiseptated lesions with variable degree of calcification. CT typically demonstrates a hypodense, large lesion with walls having minimal or no enhancement (Fig. 3). In case of detaching endocyst, the detached wall appears as an undulating structure. Daughter cysts may also be visible at different stages of the disease and when present they are pathognomonic. The presence of hypointense wall on both T1- and T2-weighted images is characteristic for cystic hydatid disease. Contrast enhancement of the cyst is typically not detected on either CT or MR images, a finding that may serve as a helpful feature to differentiate degenerated hydatid cysts from cystic biliary cystadenomas/cystadenocarcinomas.

**Fig. 4:**
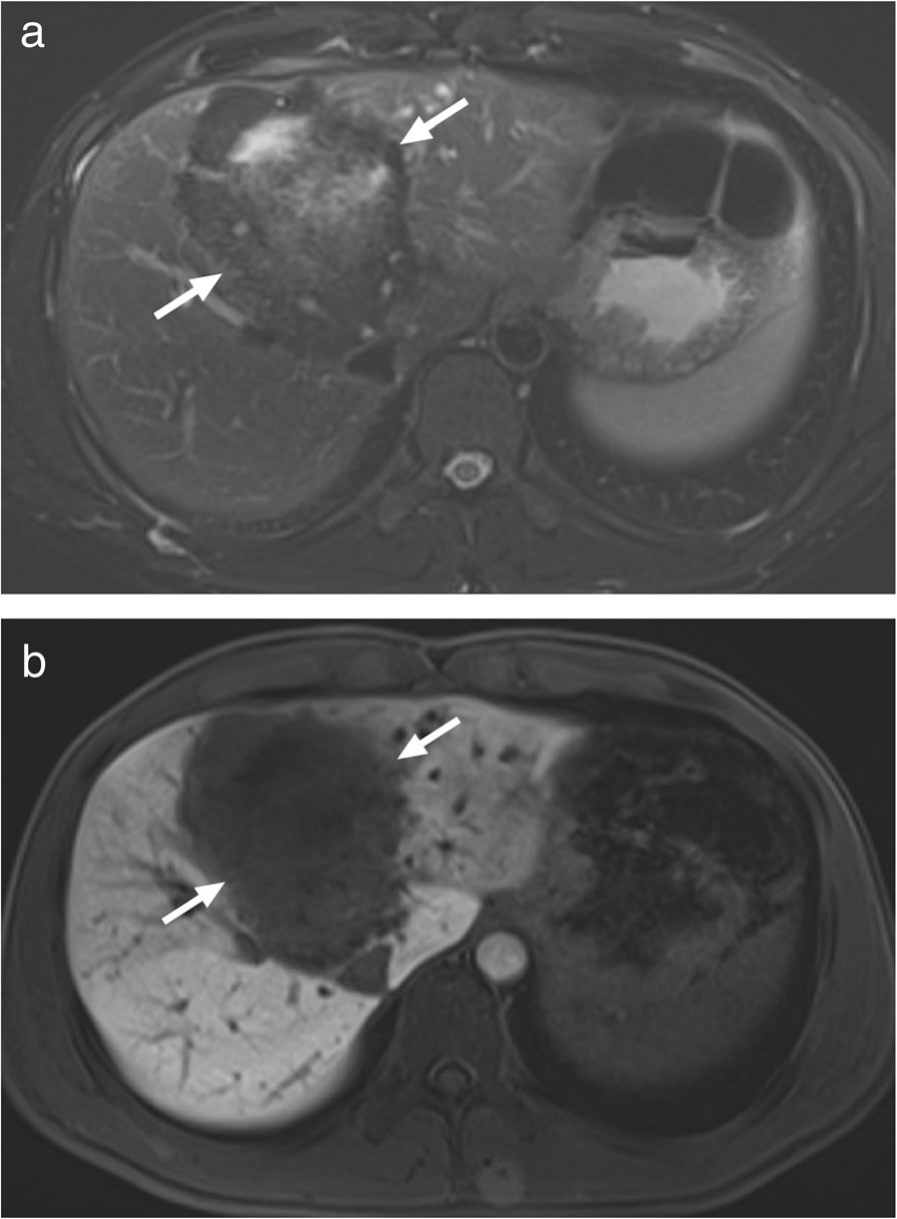
A 27-year-old male patient with no known past medical history presented with jaundice and right upper quadrant pain. **a** Axial plane T2W image demonstrates a large, mostly hypointense mass with lobulated borders (arrows). **b** Axial plane postcontrast hepatobiliary phase T1W fatsat image better demonstrates the borders of this mass (arrows). Percutaneous biopsy revealed alveolar hydatid cyst

**Fig. 5:**
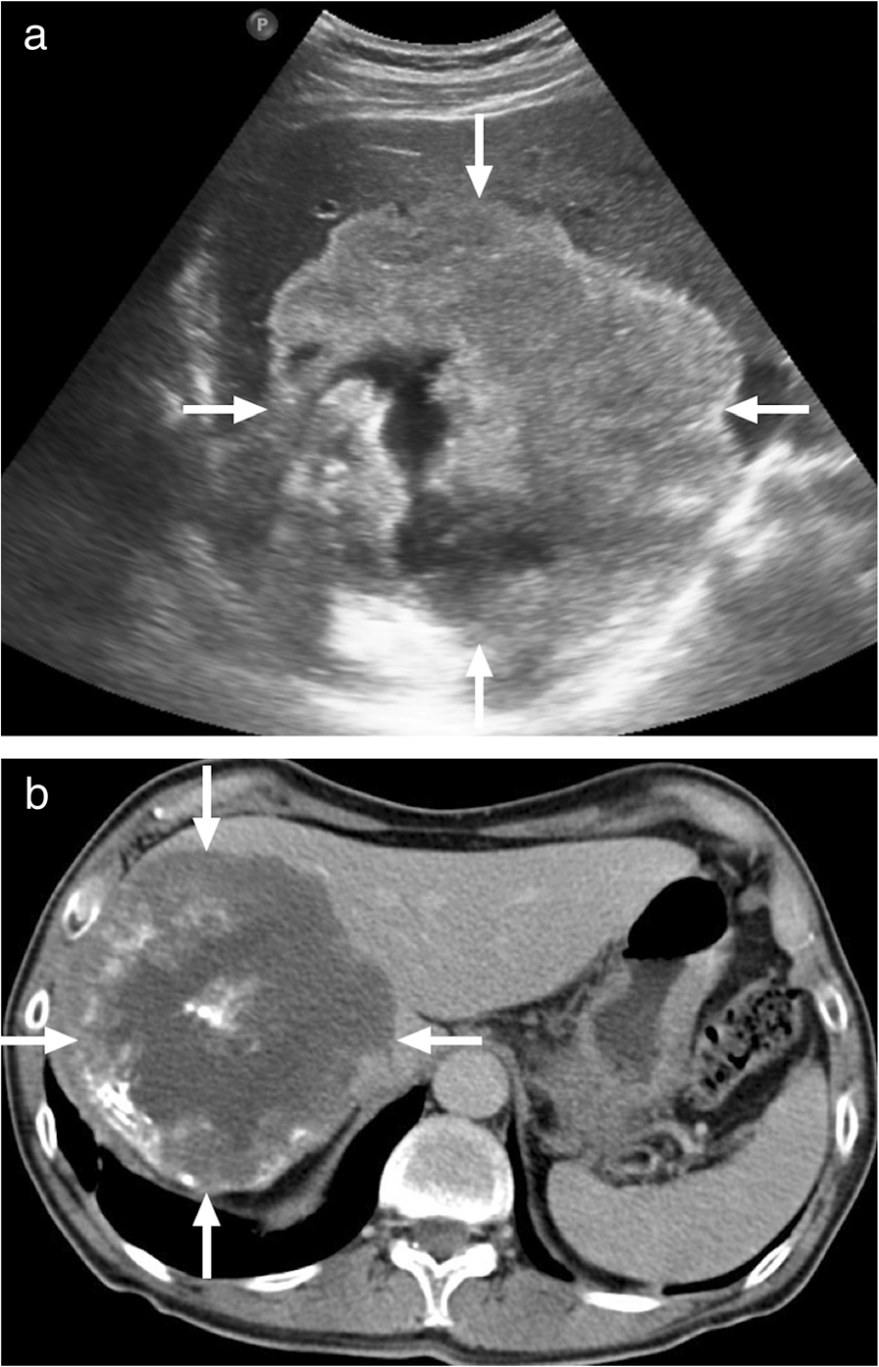
A 65-year-old male recently presented with right upper quadrant pain and abnormal liver function tests. **a** Abdominal ultrasound demonstrates lobulated, centrally cystic mass (arrows) within the right liver lobe. The mass was mostly hyperechoic in echotexture. **b** Axial plane postcontrast CT demonstrated a large, hypoattenuating, centrally cystic/necrotic liver mass (arrows) with scattered calcifications. Percutaneous biopsy was performed to exclude cholangiocellular cancer. Final diagnosis was alveolar hydatid cyst. The patient underwent right hepatectomy

**Fig. 6:**
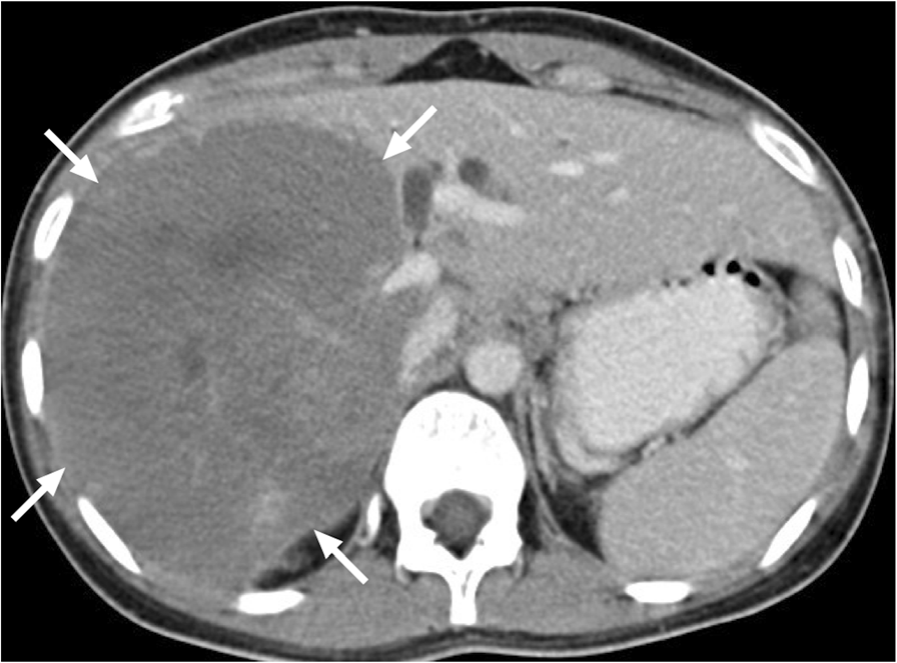
A 33-year-old female with no known past medical history presented with gradually increasing right upper quadrant pain. Abdominal postcontrast CT demonstrates a hypoattenuating centrally necrotic massive mass lesion (arrows). There was no evidence of obvious enhancement after contrast injection. Percutaneous biopsy revealed alveolar hydatid cyst

*E*. *multilocularis* infection, the alveolar form of hydatid disease, may be more difficult to differentiate from malignant lesions of the liver. The disease is biologically aggressive and typically presents as a solid, infiltrative heterogenous mass with indistinct borders (Fig. 4). There may be small cysts. Nodular and dystrophic calcifications may be detected (Fig. 5) [1]. On CT, the mass also typically includes hypoattenuating areas corresponding to necrosis and parasitic tissue. In certain cases, *E*. *multilocularis* infection may simulate a large hemangioma or a cholangiocellular carcinoma. There may be mild delayed peripheral enhancement, which corresponds to the fibroinflammatory peripheral rim, in both postcontrast CT and MRI studies (Fig. 6) [19]. On MRI, the lack of diffusion restriction in the mass is a helpful finding for differentiating alveolar echinococcosis from malignant neoplasms such as cholangiocarcinoma [19]. Familiarity with the characteristic imaging findings of alveolar echinococcosis with supportive serologic and clinical information is essential for diagnosis in patients from endemic regions.

Percutaneous treatment may be successfully used for treatment of *E*. *granulosus* infection but surgery is typically indicated for *E*. *multilocularis* infections. The most fearsome potential complication of percutaneous treatment of hydatid cyst is anaphylactic reaction. Despite the fact that it is rare, it can be fatal and therefore the interventionalist should take relevant precautionary measures before starting the procedure [20].

### Pyogenic and amebic liver abscesses

**Fig. 7:**
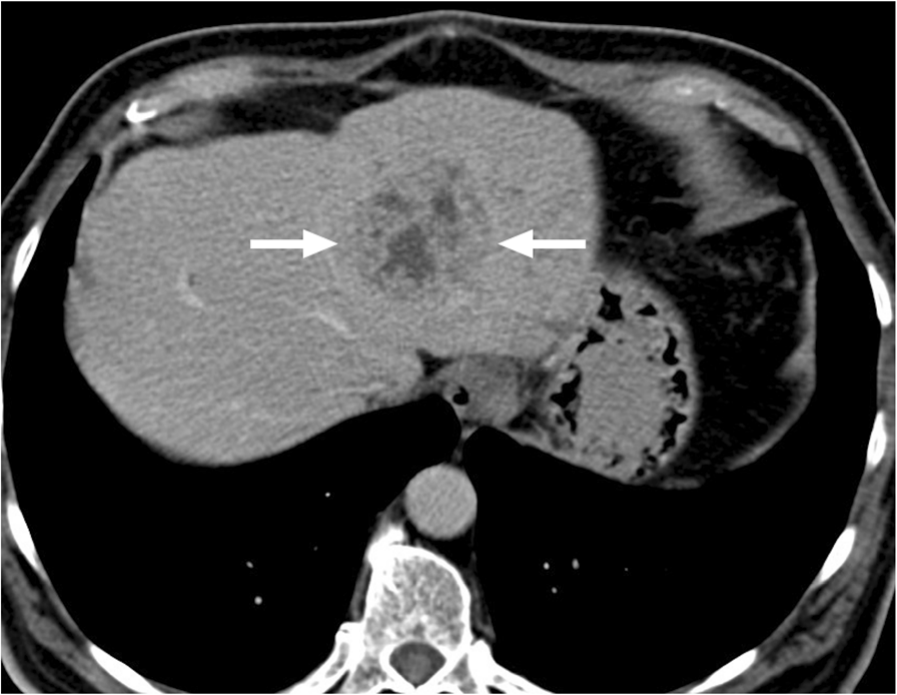
A 70-year-old male with known colon cancer under complete remission underwent a routine screening CT examination. Axial plane postcontrast CT image revealed 5 cm predominantly solid mass (arrows) within the left liver lobe. The patient was completely afebrile and free of any abdominal pain at the time of the CT scan. Percutaneous biopsy confirmed liver abscess with no evidence of neoplastic cells. Follow up CT scan (not shown) after IV antibiotics confirmed significant regression of the lesion

**Fig. 8:**
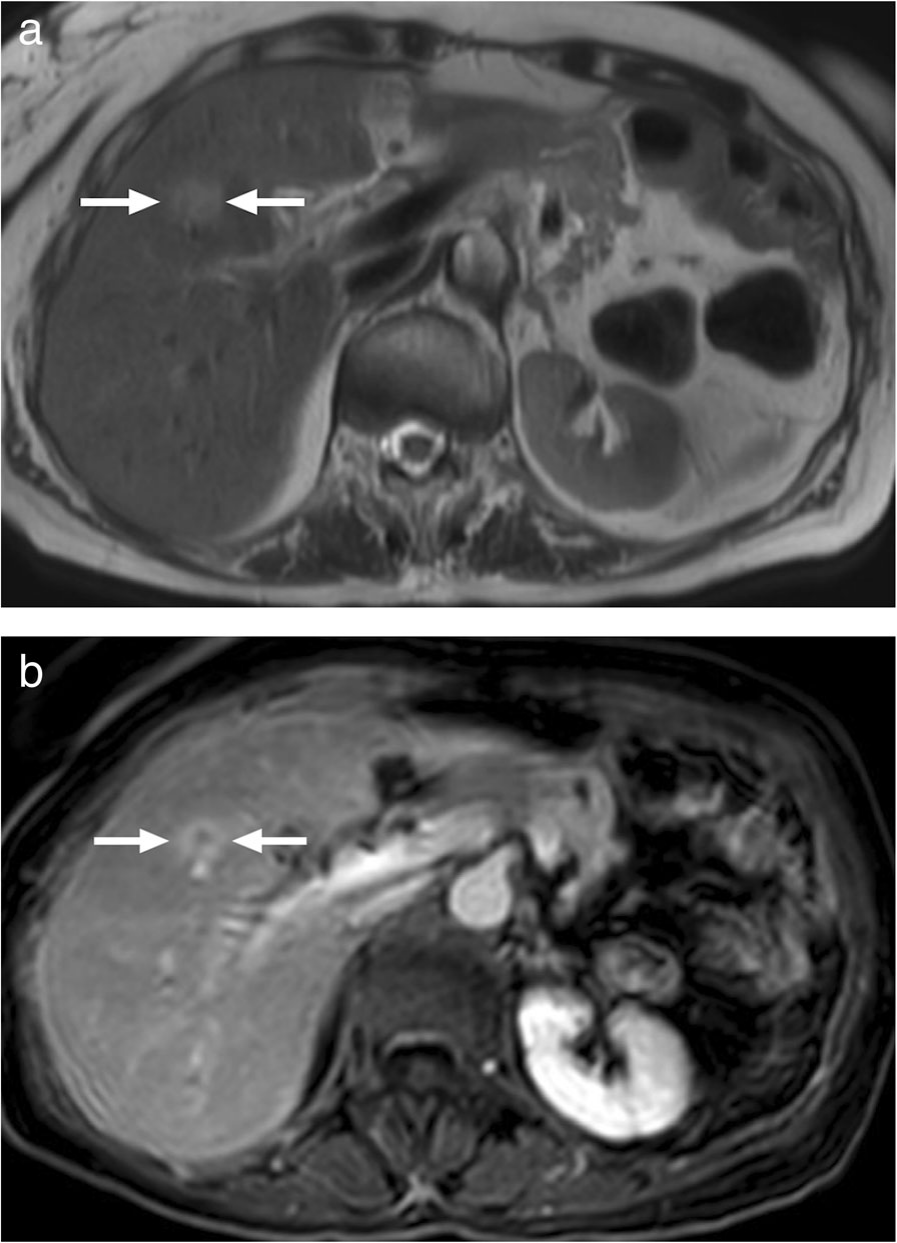
A 64-year-old female with previously surgically treated pancreatic adenocarcinoma now presenting with a hypoechoic focal liver lesion detected in outside US. Clinically the patient was in complete remission for her tumor and her prior abdominal scan was normal. She was asymptomatic and had no fever. **a** Axial plane T2W MR image demonstrates a moderately hyperintense focal lesion (arrows) in the right liver lobe. **b** Axial plane postcontrast T1W MR image at the late arterial phase demonstrates marked ring enhancement in the periphery of the lesion (arrows) which was considered to be highly suspicious for an adenocarcinoma metastasis. Percutaneous biopsy did not demonstrate any neoplastic cells but was interpreted as an abscess. Follow up CT scan (not shown) after intense IV antibiotic treatment showed complete disappearance of the lesion

Hepatic abscesses may be secondary to fungal, bacterial, or amebic infections. Pyogenic abscesses represent the vast majority of these abscesses with *Escherichia coli* and *Clostridium* species being the common causative agents [21]. Solitary hepatic abscesses are often cryptogenic with no clear-cut source to be identified [22, 23]. CT typically shows a hypoattenuating cystic lesion with surrounding parenchymal edema (Fig. 7). This parenchymal edema is a very useful sign for differentiating pyogenic abscesses from cystic metastases. However, it should also be noted that some solitary liver abscesses may present as mostly solid lesions with no identifiable underlying risk factor. Without perilesional edema and typical septations, liver abscess may be difficult to recognize (Fig. 8). Also, the presence of fever is not a universal feature of liver abscesses, further complicating diagnosis. The absence of centrally progressing contrast enhancement on delayed phase images and progressive enhancement in the outermost layer corresponding to the fibroinflammatory capsule of the abscess may be helpful for differentiating mostly solid appearing pyogenic abscesses from intrahepatic cholangiocarcinomas. It is also difficult to differentiate necrotic liver tumors from abscesses. Nodularity in the internal surface of the lesion wall, focal calcifications within the lesion, and the absence of perilesional edema favors necrotic neoplasm over an abscess [24]. Follow-up imaging after empirical treatment for liver abscess may also be implemented as an alternative approach for differential diagnosis. However, morphological changes may also be observed during the natural course of the disease without any therapeutic intervention. In patients where no diagnosis could be achieved in a noninvasive manner, percutaneous biopsy may be utilized for definitive treatment planning.

**Fig. 9:**
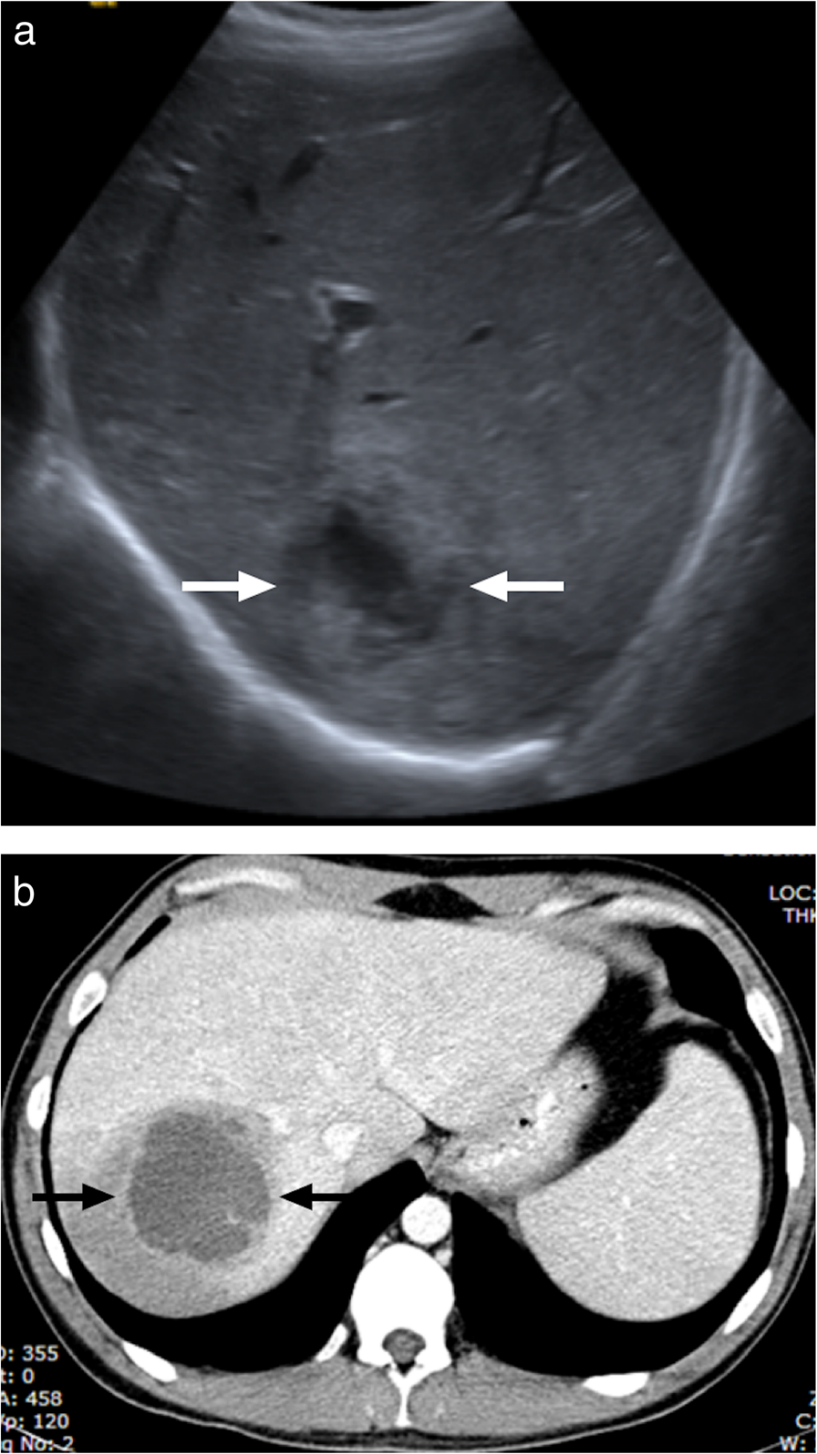
A 32-year-old male with no known medical history now presented with right upper quadrant pain and fever. His personal history was remarkable for a recent trip to India. **a** Sonographic study revealed semisolid mass located within the posterior segment of the right liver lobe. **b** Axial plane postcontrast CT image demonstrates thick-walled, centrally necrotic mass lesion (arrows) with peripheral hypodense area consistent with edema. Percutaneous sampling revealed amebic liver abscess

Amebic abscesses are caused by the protozoan *Entamoeba histolytica* [21]. In around 70% of the cases, a solitary abscess within the right liver lobe is detected. US typically shows a round or oval shaped, hypoechoic lesion without any significant wall echoes [25]. On contrast enhanced CT, amebic abscess usually may appear as round shaped, hypoattenuating lesions with ill-defined borders (Fig. 9). An enhancing wall and a peripheral edema are also a common feature. Also, septations may be seen within the abscess cavity [24, 26].

### Eosinophilic liver abscess

**Fig. 10:**
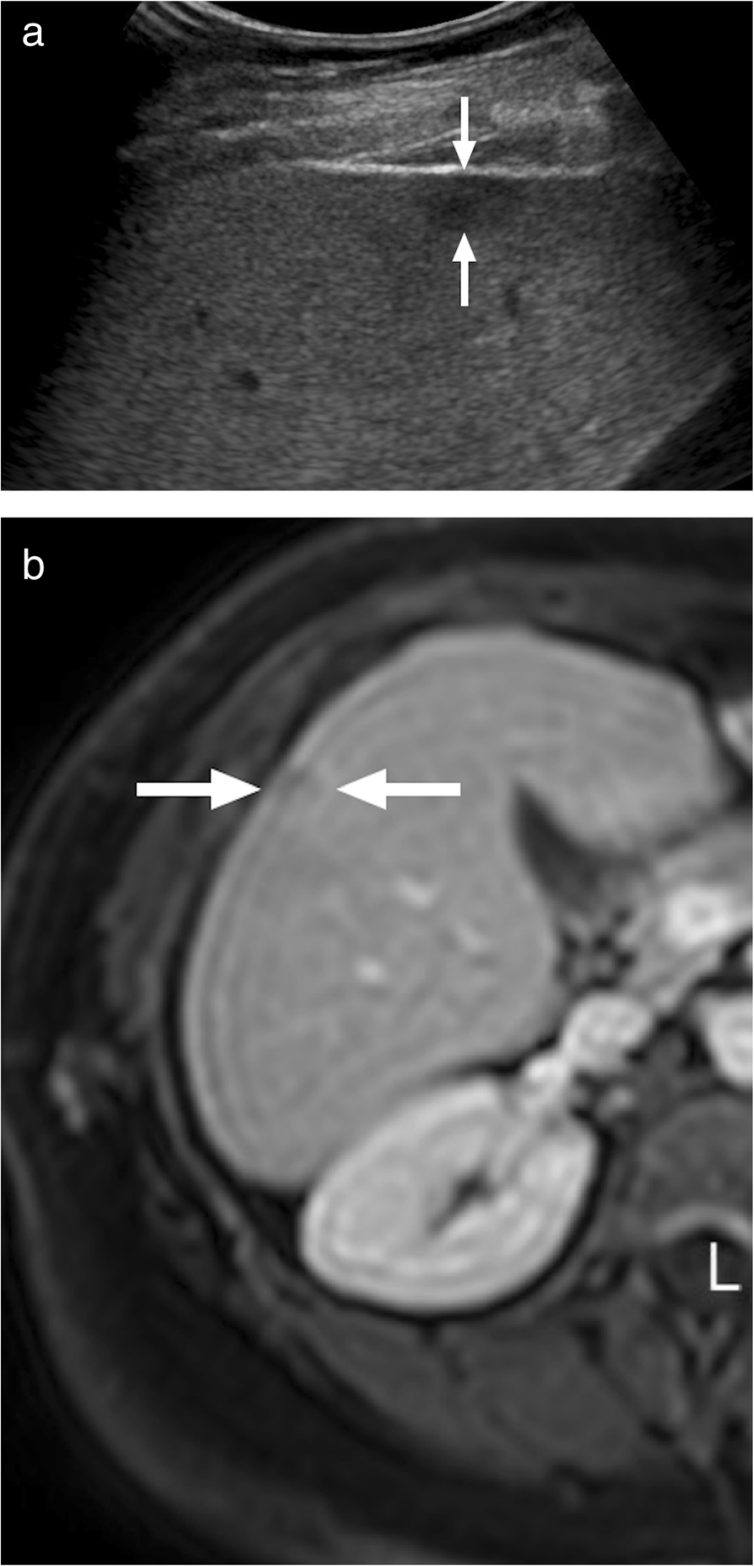
A 50-year-old female with known breast cancer in remission was referred to MRI study after detection of a subcentimeter focal liver lesion on US exam. **a** US study showed subcapsular, hypoechoic solid appearing mass (arrows). **b** Axial plane postcontrast T1W image showed the same lesion as a hypointense mass with peripheral contrast enhancement (arrows). DW images and ADC map confirmed significant diffusion restriction (not shown). Percutaneous biopsy confirmed eosinophilic liver abscess with no evidence of metastatic disease. Subsequent peripheral blood smear also confirmed hypereosinophilia

Eosinophilic liver abscess (ELA) consists of focal eosinophil-related parenchymal liver necrosis. Hepatic eosinophilia and hepatic eosinophilic granuloma are alternative terms for this clinical entity. Most cases are incidentally diagnosed in asymptomatic patients who also typically have peripheral eosinophilia. The disease usually runs a benign course. Hepatic migration of parasitic larvae has been implicated in the etiology; however, medications, allergic, and neoplastic diseases have also been considered in the pathogenesis [27]. This is a rare clinical condition and may mimic liver metastases on imaging studies (Fig. 10) [28].

Typically, ELAs are seen as multiple small, hypodense, and oval-shaped lesions on CT [27]. Multifocality and a history of parasitic disease may be helpful for differential diagnosis. Follow-up imaging may also show regression of the liver lesions which may be helpful for differential diagnosis. However, definitive diagnosis almost always requires histopathologic confirmation [29].

### Fascioliasis of the liver

**Fig. 11:**
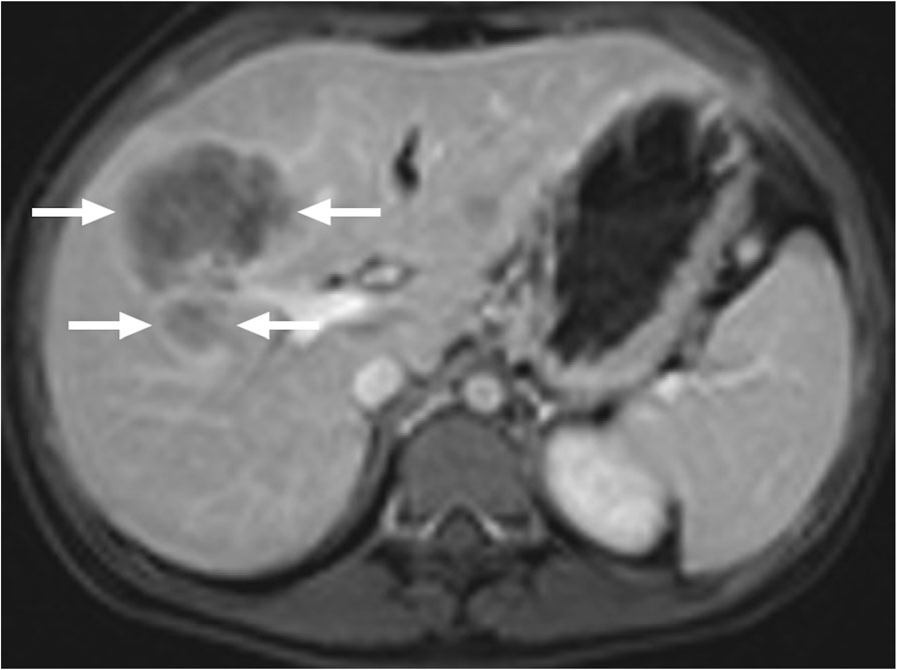
A 16-year-old girl with known surgically treated Wilms tumor undergoing MRI evaluation for newly detected hypoechoic liver mass on US. Axial plane postcontrast T1W MR image demonstrates two peripherally enhancing lesions (arrows) in the right liver lobe. Post percutaneous biopsy showed fascioliasis with no evidence of malignant cells

Fascioliasis is an infection caused by *Fasciola hepatica* flatworm. The larvae are acquired by ingesting infected water. The parasites may stay dormant within the liver parenchyma for years and this chronic presence typically leads to chronic parenchymal inflammation [30]. The typical imaging findings include tunnel-shaped microabscesses and necrotic parenchymal cavities along the migration tract of the larva. These tunnels often extend from Glisson’s capsule to the central portions of the liver [30]. Peripheral eosinophilia is a common finding in fascioliasis and may be helpful to reach a correct diagnosis [31]. Imaging presentation may mimic malignant processes in some patients (Fig. 11). Histopathologic diagnosis may be necessary in these patients for correct diagnosis, particularly without antecedent imaging findings that show tunneling tracts.

### Rare granulomatous liver infections

**Fig. 12:**
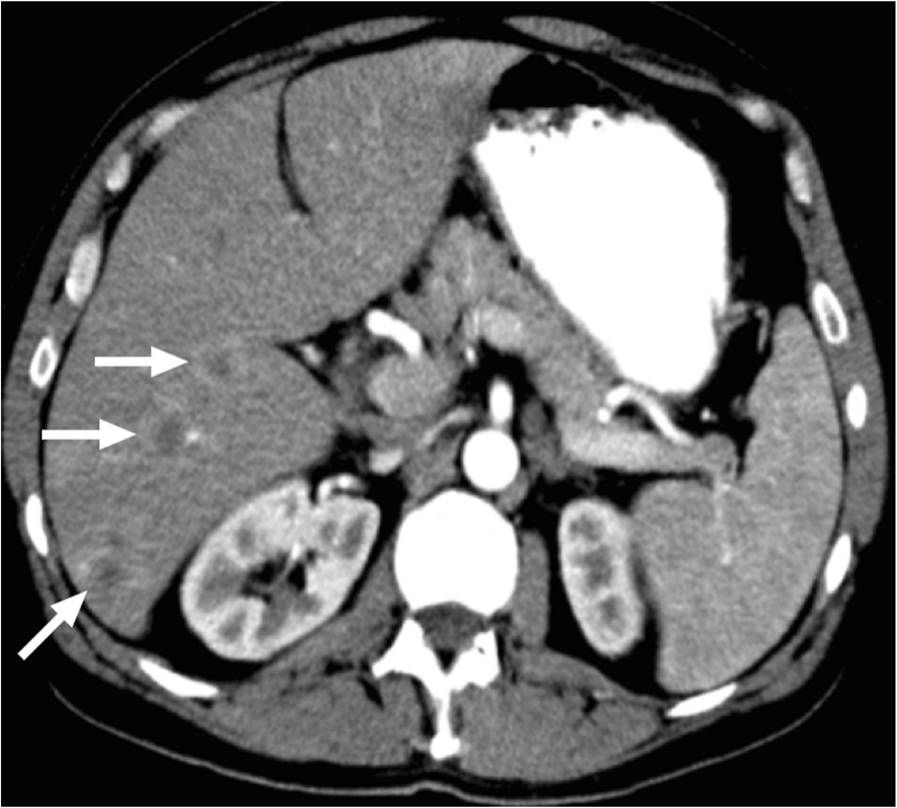
A 48-year-old male with known HIV presenting with recent onset of abdominal pain and mild weight loss. Axial plane postcontrast arterial phase CT image demonstrates several peripherally enhancing liver lesions (arrows) located within the right lobe. Metastatic liver disease or pyogenic microabscesses were initially considered. Percutaneous biopsy was consistent with syphilitic liver abscesses. The RPR test was also found to be positive after the biopsy diagnosis

Spirochetal infections of the liver are rare in the post-antibiotic era [32]. Spirochetal hepatitis can lead to cirrhosis, and the detection of gummas, caseating, and non-caseating granulomas and focal necrosis around the hepatic veins are characteristic histologic findings [33, 34]. Liver involvement may be seen in secondary and tertiary stages of syphilis. Focal gumma may develop in the liver typically 1–10 years after the original infection [35]. There is not much information in the imaging literature due to the rarity of hepatic involvement. However, it appears that low attenuation lesions with peripheral enhancement on CT (Fig. 12) are a potential finding. Calcification may also be rarely present [36, 37].

**Fig. 13:**
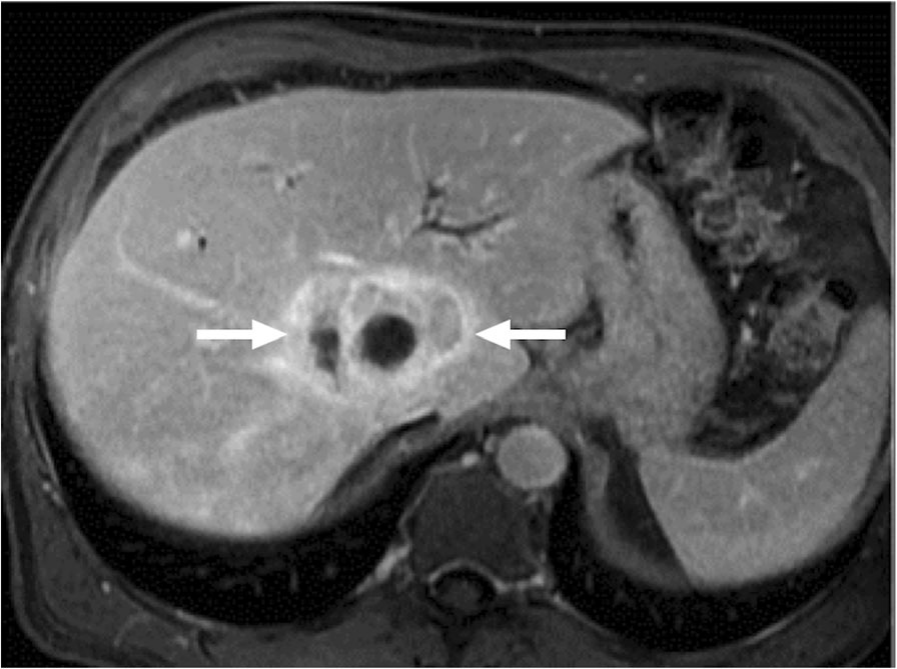
A 44-year-old male, previously healthy professional gardener on the island of Martha’s Vineyard, now presenting with cutaneous ulcer and lymphadenopathy on his arm, right upper quadrant pain, fever, and malaise. He was referred to MRI for a mass within the liver detected on abdominal US. Axial plane postcontrast image demonstrates an avidly enhancing, predominantly solid mass (arrows) adjacent to inferior vena cava and the caudate lobe. Acute titers for tularemia were elevated. Titers normalized and the lesion almost completely resolved on follow up MRI after doxycycline treatment

*Fransicella tularensis* is a Gram-negative coccobacillus endemic to many parts of North America. The most common presentation is the ulceroglandular form. Untreated disease can spread to multiple organs. Liver involvement is common and may be observed in 75% of cases with extralymphatic organ involvement [38]. The spectrum of findings in tularemia is considered to be in the spectrum of granulomatous hepatitis [39]. Visible hepatic abscess can form, which in our experience can appear quite solid on CT (Fig. 13).

**Fig. 14:**
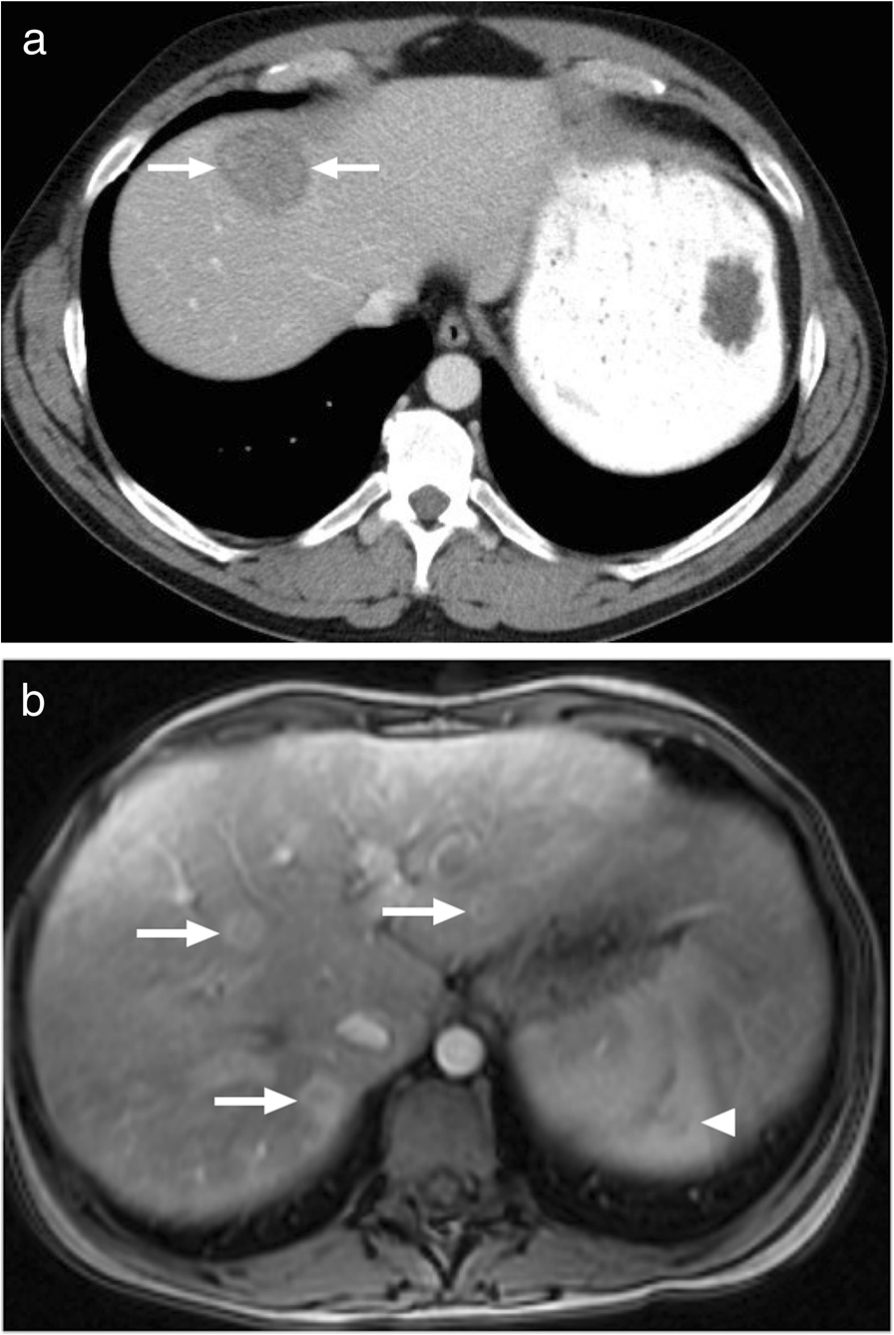
Images of two different patients with pathologically proven hepatic Bartonella granulomas. **a** Axial plane postcontrast CT image of a 45-year-old male demonstrates predominantly hypodense solid mass (arrows), with central heterogeneity, located in the liver dome. Surgical resection of this lesion confirmed Bartonellosis. **b** A 13-year-old girl presented with abdominal pain and referred for MRI with liver lesions detected on ultrasound. Axial plane postcontrast arterial phase image demonstrates avidly ring-enhancing liver masses (arrows) and similar lesions in the spleen (arrowhead) which were more evident on portal venous phase image (not shown). These lesions were concerning for hypervascular metastasis. Acute titers were positive for Bartonella and the patient recovered uneventfully

Bartonellosis is among the causes of fever of unknown origin and unusual presentations may happen in 0.3–10% of cases [40]. In cases with hepatic and splenic involvement, granulomatous inflammatory process is typically the hallmark pathologic finding. Imaging findings are non-specific but hypoenhancing or non-enhancing focal parenchymal lesions may be observed (Fig. 14a). MR imaging features are not well described, with only anecdotal case reports. T2 hyperintensity and postcontrast enhancement on T1W images are among the reported findings (Fig. 14b). Calcifications may appear within these nodules on long-term follow-up [3, 41]. The nodules may easily be confused on CT and MR with hypoenhancing metastases. It should also be noted that the disease may appear as a single mass within the liver parenchyma mimicking a primary hypoenhancing cholangiocellular carcinoma.

### Actinomycosis of the liver

**Fig. 15:**
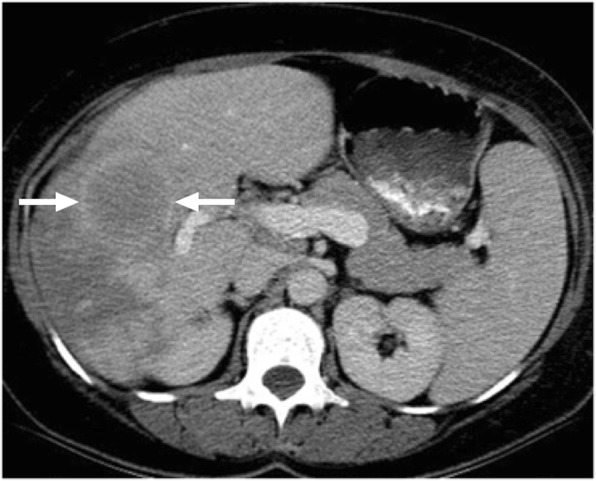
A 40-year-old female presenting with mild fever and liver mass on US examination. Axial plane postcontrast CT image demonstrates ill-defined, hypoattenuating mass (arrows) in the right lobe with accompanying wide parenchymal area of disturbed perfusion. Percutaneous biopsy revealed basophilic aggregates and sulfur granules consistent with actinomycetes infection. Post-antibiotic treatment demonstrated almost complete regression of the imaging findings (not shown)

**Fig. 16:**
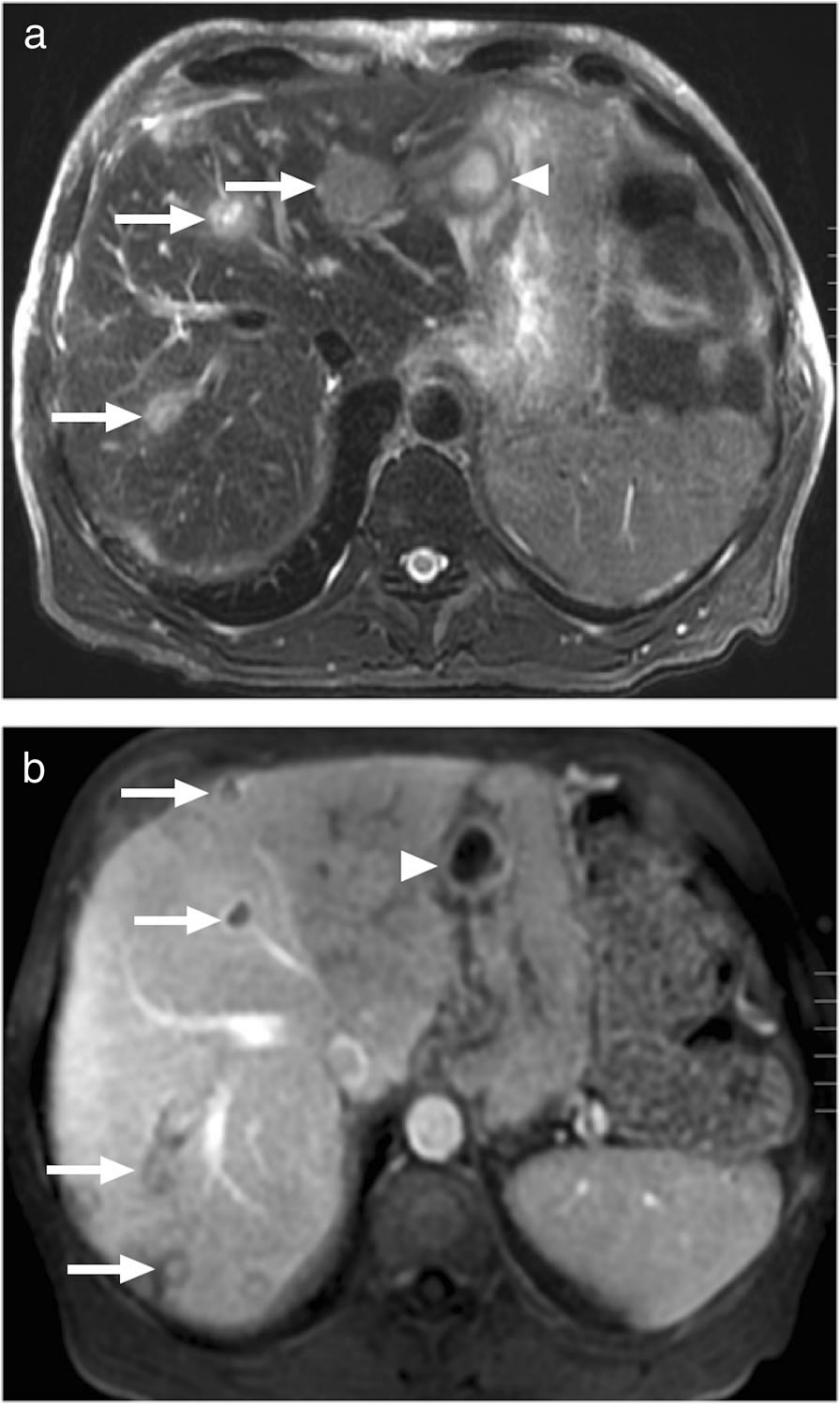
A 66-year-old male with poorly controlled diabetes presented with abdominal pain, fever and weight loss. **a** Axial plane T2W MR image demonstrates hyperintense, predominantly cystic lesions (arrows). Also note is made of necrotic lymph node in the gastrohepatic ligament (arrowhead). **b** Axial plane postcontrast T1W MR image demonstrates intense peripheral contrast enhancement (arrows). Percutaneous biopsy confirmed actinomyces infection.

Actinomyces are slow growing, Gram-positive, branching bacilli. They are most frequently associated with infections in the cervicofacial region. Abdominal involvement is rare, where iliocecal area is the most frequently affected region. Hepatic infection has been reported to be seen in 15% of those with abdominal infection and, overall, represents 5% of all cases with actinomycosis [42]. Imaging studies may reveal multiple or solitary lesions, a single hypoattenuating mass the most common presentation (Fig. 15) [43]. T1 hypointensity, with variable contrast enhancement, and associated T2 hyperintensity are the commonly encountered findings on MRI studies (Fig. 16) [44].

### *Pneumocystis jirovecii* infection of the liver

**Fig. 17:**
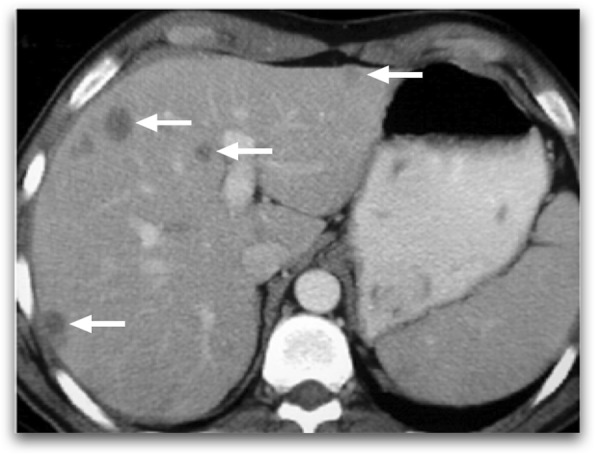
A 55-year-old male with newly diagnosed HIV infection who presented with high fever and generalized abdominal pain. His CD4^+^ lymphocyte count was also significantly low at the time of his presentation. Axial plane postcontrast CT scan revealed several small hypoattenuating lesions scattered throughout the liver parenchyma (arrows). Based on his clinical context, the lesions were suggestive of pneumocystis jirovecii infection. Also note mild splenomegaly in this case which is an important suggestive finding for disseminated pneumocystis infection. Post treatment follow up confirmed almost complete regression of the imaging findings (not shown)

*Pneumocystis jirovecii* is a common opportunistic infection in immunocompromised and HIV-infected patients and lung is the most commonly involved organ. Liver involvement is not common, but when present, hypodense lesions within the liver and spleen may be seen on CT (Fig. 17). The differential diagnosis from a neoplastic process is even more difficult when infection is the presenting symptom of unrecognized retroviral infection. In our limited experience, lesions are almost always multiple rather than solitary. Clinical and medication history as well as the immune status of the patient are key factors for the correct diagnosis. The presence of associated lung findings may also be helpful.

### Sarcoidosis

**Fig. 18:**
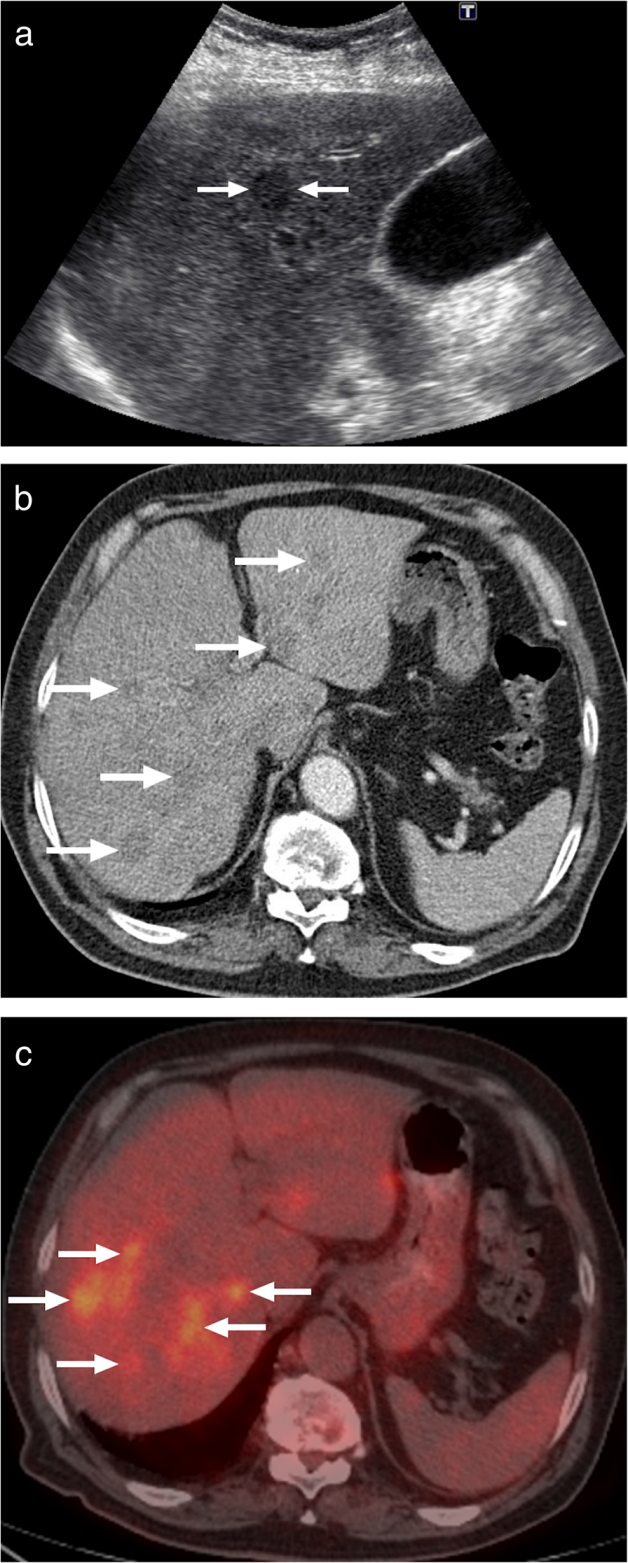
A 60-year-old male with previously treated lymphoma, in complete clinical and imaging remission, now presents with fatigue, fever, and malaise. **a** Abdominal US image demonstrated several hardly discernible subcentimeter lesions throughout the liver parenchyma. Only one lesion was larger than a centimeter (arrows). **b** Axial plane postcontrast CT image demonstrates innumerable hypodense lesions (arrows). **c** On PET-CT, these lesions were found to be metabolically active with intense FDG uptake (arrows). Chest and neck CT studies were unremarkable (not shown). Percutaneous biopsy surprisingly showed noncaseating granulomas consistent with sarcoidosis. Symptoms and imaging findings promptly resolved after corticosteroid treatment

Sarcoidosis is a systemic granulomatous disease. The exact etiology is obscure and the detection of non-caseating granulomas on pathology specimens is characteristic for this disease. Lungs are the most commonly involved organs but virtually any organ can be affected in the course of the disease [45]. Liver is the third most commonly affected organ after lung and lymph nodes and in most cases hepatic involvement occurs in the course of systemic disease. However, isolated liver involvement had also been reported [45]. The detection of liver involvement may be delayed as most of the affected patients are asymptomatic [46]. Although the liver is commonly involved, in only 5% of sarcoid cases will liver lesions be detected with CT [47]. Liver involvement in sarcoidosis may present in diffuse or focal nodular patterns [46]. Focal lesions are typically small and randomly scattered throughout the liver parenchyma. On CT, the lesions are mostly hypoattenuating without significant contrast enhancement (Fig. 18). MR imaging may also be helpful for lesion detection. Concurrent splenic lesions should be sought, as these are often more readily detected than sarcoid in the liver. From an imaging standpoint, differentiation from metastatic disease, lymphomatous involvement, or other infectious processes may be difficult without relevant clinical and serologic information.

Elevated serum ACE levels are a relatively common finding which may act as a supportive information to reach the diagnosis. Despite best efforts, image-guided percutaneous biopsy may be necessary for definitive diagnosis [45].

### IgG4-related focal liver disease

**Fig. 19:**
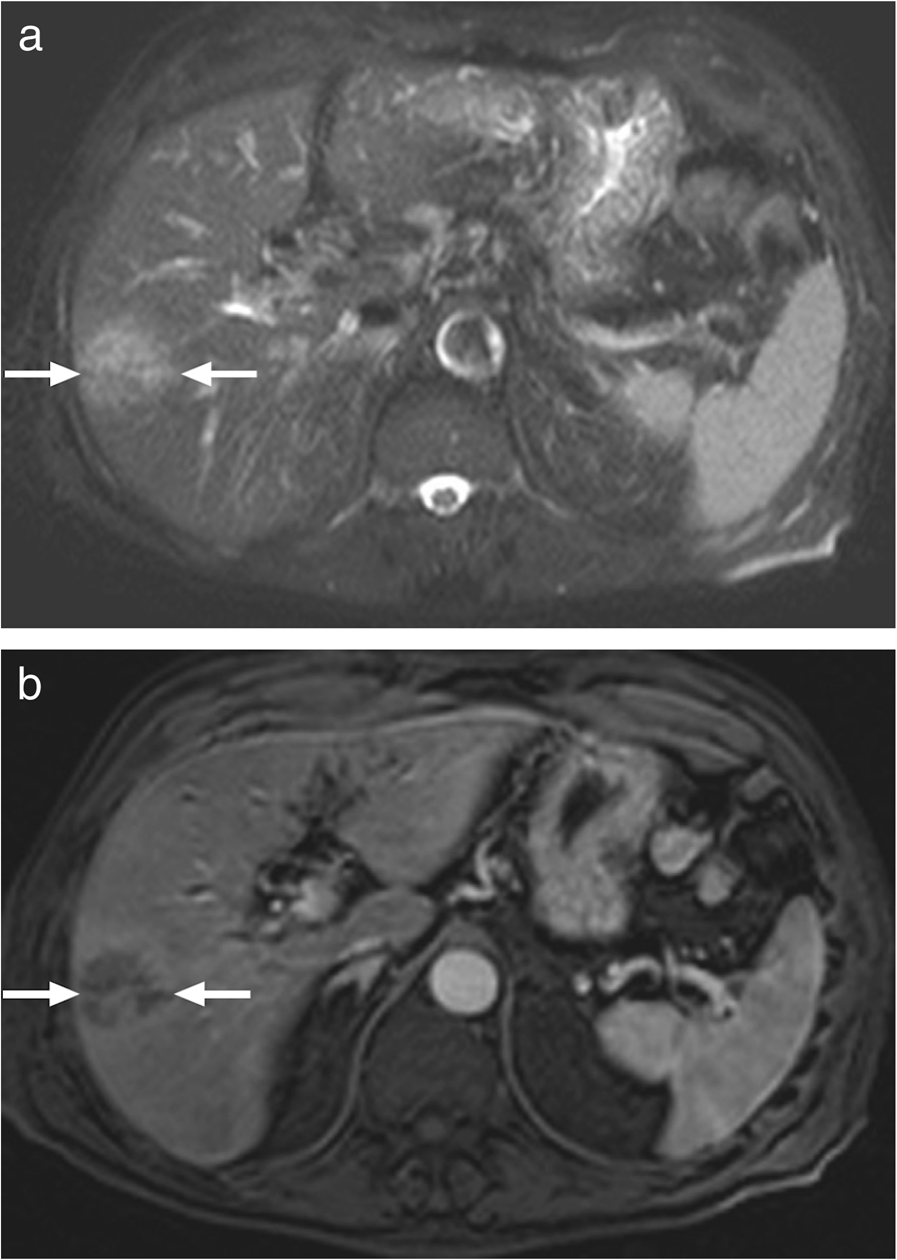
A 70-year-old male with no significant past medical history presenting with focal hypoechoic liver mass on an US exam performed for new onset right upper quadrant pain. **a** Axial plane T2W image demonstrates moderately hypointense mass (arrows) in the right liver lobe. **b** Same lesion demonstrates mild peripheral enhancement with no significant central contrast filling (arrows). The putative diagnosis was cholangiocellular carcinoma. Post biopsy pathologic examination confirmed IgG4-related focal liver pseudomass with no evidence of neoplastic cells

**Fig. 20:**
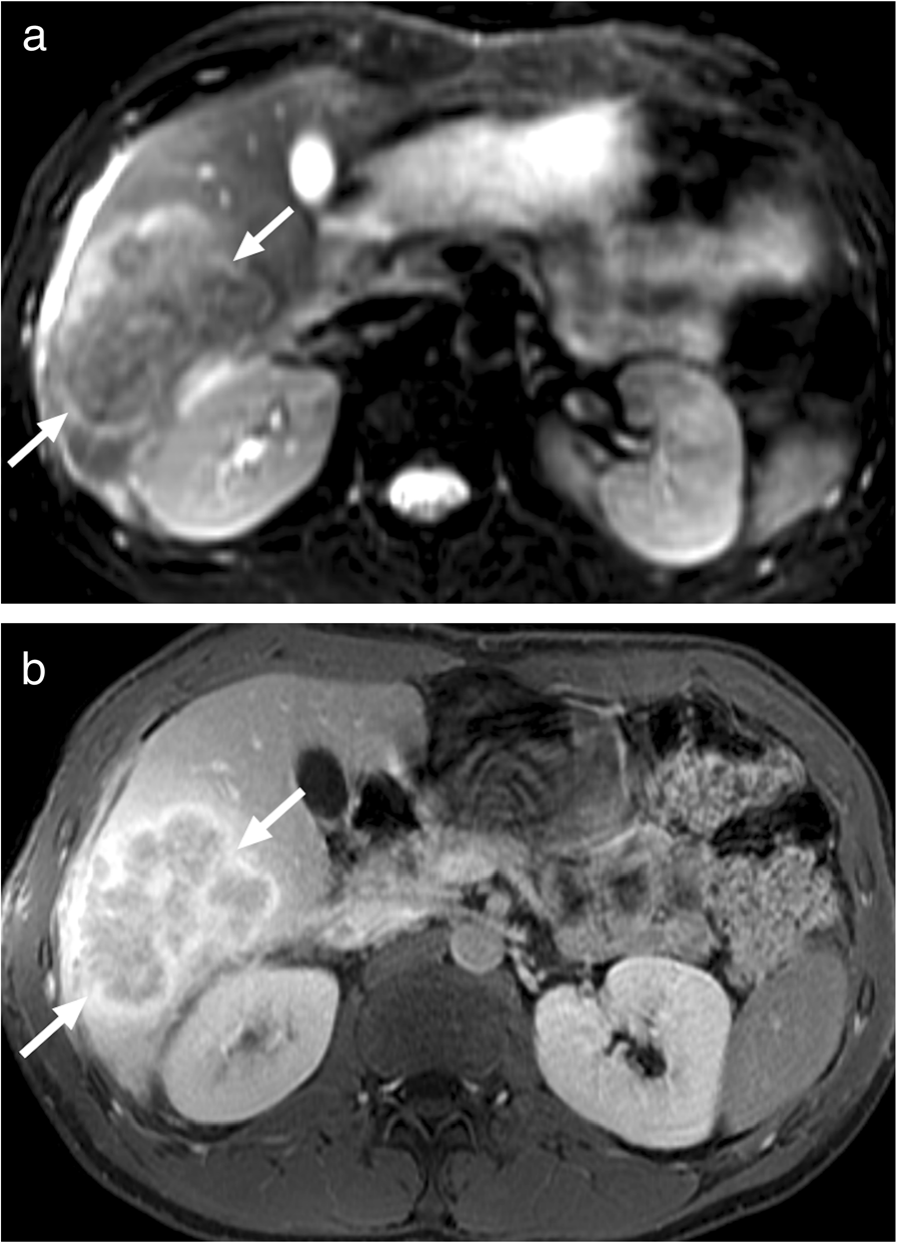
A 28-year-old male with no significant medical history presented with right upper quadrant pain and weight loss. **a** T2W axial plane MR image demonstrates a solid mass (arrows) within the right liver lobe. **b** Postcontrast axial plane T1W image in the portal venous phase demonstrates heterogenous enhancement of the lesion (arrows). Surgical resection, with a putative diagnosis of cholangiocellular carcinoma, of this mass revealed inflammatory pseudotumor with features suggestive of IgG4-related sclerosing cholangitis. Post-surgical evaluation revealed elevated serum IgG4 levels

IgG4-related disease commonly affects the liver and the biliary system. Histopathologically IgG4-related hepatic disease is characterized as a fibroinflammatory condition with dense lymphoplasmocytic infiltrate rich in IgG4-positive plasma cells with or without elevated serum IgG4 levels [48]. Focal parenchymal mass with well-defined borders is relatively rare in patients with liver involvement [49]. Differentiation from liver malignancy may be extremely difficult without proper history and pathological analysis (Figs. 19 and 20) [48, 50].

### Inflammatory pseudotumor of the liver

Inflammatory pseudotumor of the liver was first described in 1953 and more than 200 patients have since been reported [51, 52]. The etiology remains largely unknown but autoimmune reactions, infectious hepatitis, and bacterial infections have all been implicated in the etiology [53]. In certain patients, differential diagnosis from neoplastic liver lesions may necessitate percutaneous biopsy and histopathologic confirmation. Inflammatory pseudotumors have been reported to simulate HCC in patients with viral hepatitis [53].

**Fig. 21:**
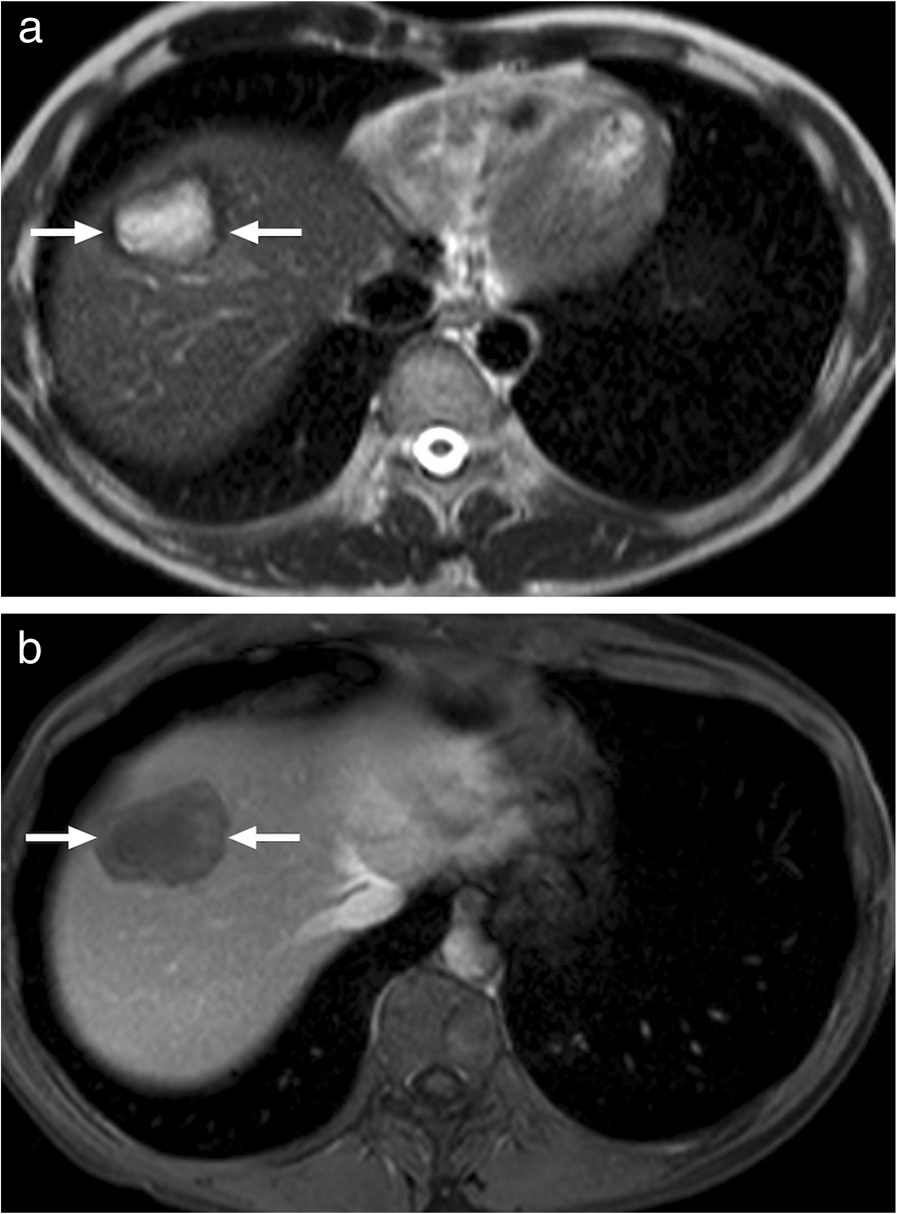
A 41-year-old male with a recently detected liver mass on US exam performed for an unrelated reason. **a** Axial plane T2W image shows a predominantly hyperintense mass with a hypointense rim (arrows). **b** Precontrast axial plane T1W image shows the rim as hyperintense (not shown). Postcontrast equilibrium phase axial plane T1W image did not demonstrate any central enhancement (arrows). As the imaging findings were not typical for any benign focal liver lesion, image-guided biopsy was performed. Pathologic examination did not reveal any neoplastic cells but was instead consistent with inflammatory pseudotumor

On US, lesions generally appear hypoechoic in the right liver lobe [54]. CT findings are highly variable. Early arterial phase enhancement mimicking an HCC or hypervascular metastasis, peripheral enhancement mimicking a cholangiocellular carcinoma or metastasis, and wash-out in the venous phase suggesting an HCC have all been reported [54, 55]. On MRI, these lesions appear hypointense on pre-contrast T1W images and iso-hyperintense on T2W images. Heterogenous or peripheral enhancement is common after contrast injection (Fig. 21) [56, 57].

### Dropped gallstones

Dropped gallstones (DS) may be seen after approximately 7% of laparoscopic cholecystectomies as a result of the rupture of gallbladder wall during dissection and removal of the gallbladder [58]. These DSs may become symptomatic in rare instances. Correct diagnosis might be extremely challenging and delayed due to atypical modes of clinical presentation and unexpected locations of these DSs [59]. Meticulous dissection and aspiration of the gallbladder during surgery comprise important factors to prevent this complication [60]. These DSs may become symptomatic with a median time interval of 5 months after the surgery, but symptoms were reported to appear even 20 years after the procedure [58].

**Fig. 22:**
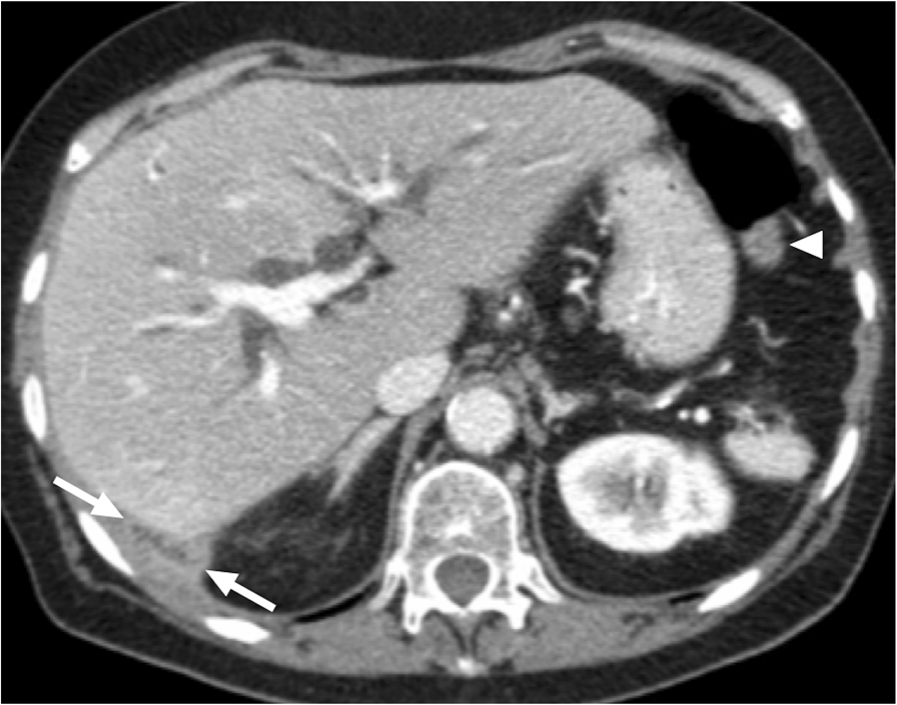
A 65-year-old male with a history of colon cancer in remission for 7 years underwent a follow up CT. He was completely asymptomatic at the time of the CT scan. He also has a remote history of laparoscopic cholecystectomy 5 years before. Axial plane postcontrast CT image demonstrates a complex looking cystic mass in the liver capsule (arrows) suggestive for a tumor implant. Also note is made of a small solid nodule in the left upper quadrant (arrowhead). Surgical excision confirmed malignant implant in the left upper quadrant and an inflammatory pseudomass in the liver capsule due to a dropped gallstone.

**Fig. 23:**
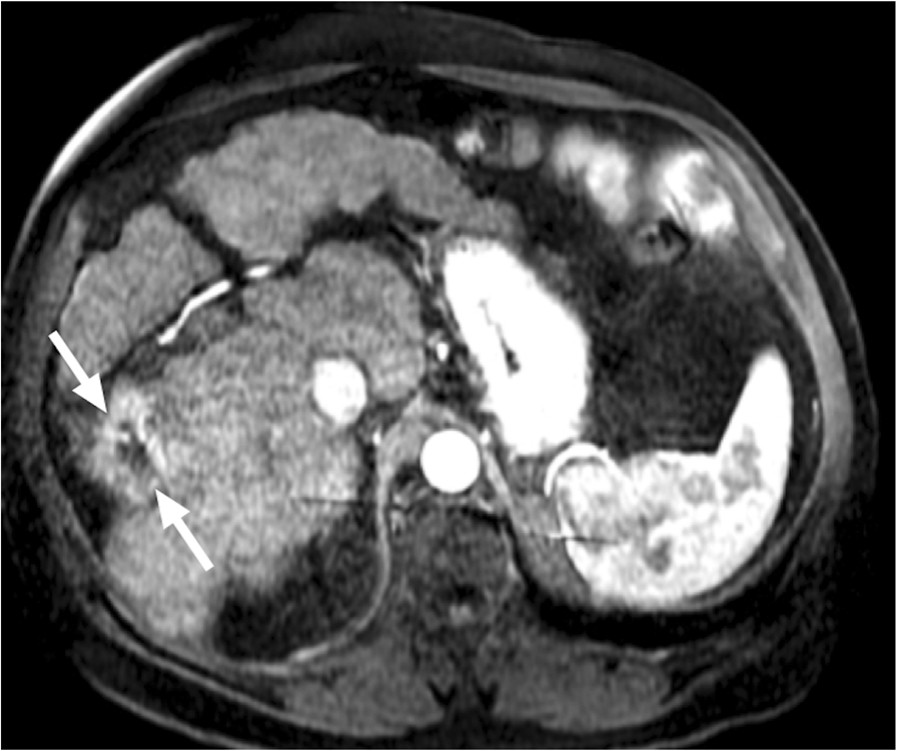
A 63-year-old male with known cirrhosis and a remote history of laparoscopic cholecystectomy underwent a screening MRI exam due to mildly elevated alpha fetoprotein. Arterial phase axial plane T1W MRI demonstrates a hyperenhancing focal parenchymal lesion (arrows) in the right lobe suggesting hepatocellular carcinoma. CT obtained to guide percutaneous biopsy revealed a small calcification (not shown), and there was a second, clearly extrahepatic collection containing well-defined dropped gallstones. Pathology specimen confirmed that the index lesion was also an abscess

Focal inflammation with abscess and fistula tract formation are relatively common complications. The detection of hyperdense structures within necrotic abscesses (in cases with dropped calcified gallstones) may be an important diagnostic clue. The location of the collection/inflammation adjacent to the liver capsule may also be regarded as a suggestive finding. Other rare complications include erosion into large bowel and bowel obstruction due to adhesion formation [58]. In patients with known malignant tumors, inflammation in hepatorenal fossa due to dropped gallstones may mimic an implant and histopathologic evaluation may be necessary (Fig. 22). Dropped gallstones may also be seen in the gallbladder fossa and differentiation from a primary focal liver malignancy may again be extremely difficult (Fig. 23).

## Gallbladder and the biliary system

Biliary dilatation and asymmetric gallbladder (GB) wall thickening are fearsome imaging findings, often indicating a pancreatobiliary system cancer or biliary stone disease. Despite these common entities, several non-neoplastic diseases may also affect the biliary system and may cause biliary system abnormalities. Primary adenocarcinoma of the gallbladder is a highly aggressive malignancy with grave prognosis. In certain patients, the gallbladder may be completely replaced by the tumor, a finding that makes difficult to conclude the source organ. However, the diagnosis may be generally made based on imaging findings. Despite this relative ease of diagnosis, one should keep in mind the several non-neoplastic mimickers of this cancer including xanthogranulomatous cholecystitis [61].

### Gallbladder-associated pseudomasses

Adenocarcinoma of the gallbladder is one of the deadliest hepatobiliary cancers. Some small cancers are incidental discoveries at cholecystectomy. When diagnosed with GB cancer by imaging, most patients are at an advanced stage with local lymph node metastases and parenchymal liver invasion. For these reasons, correct diagnosis is of critical importance and potential mimics should be considered.

**Fig. 24:**
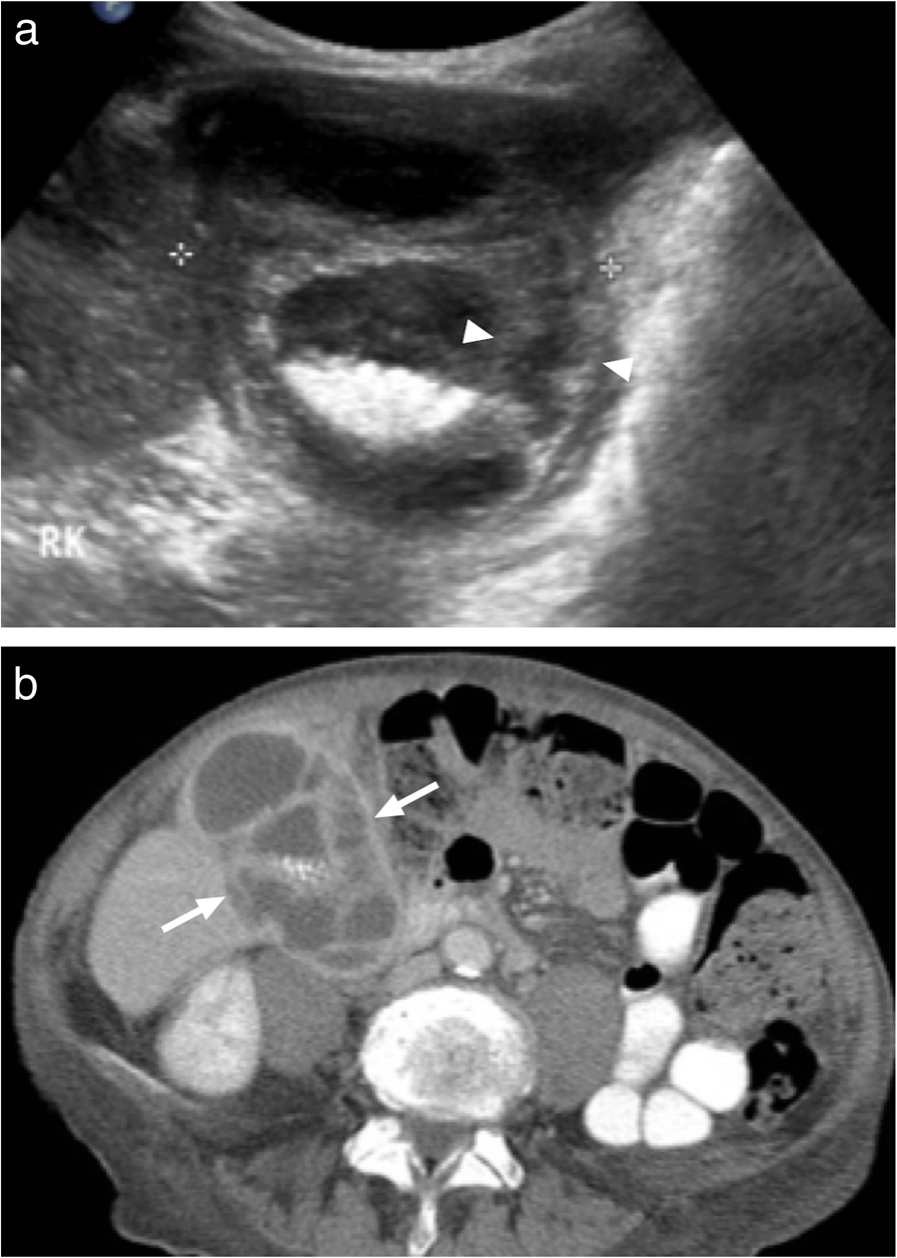
A 45-year-old male presenting to emergency room with mild fever and right upper quadrant pain. **a** US image shows significant and irregular gallbladder wall thickening (arrowheads) and hyperechoic luminal debris. **b** Postcontrast axial plane CT image demonstrates gallbladder wall thickening with intramural abscesses mimicking a multicystic mass (arrows) within the gallbladder fossa as well as intense contrast enhancement within the lesion wall. Open cholecystectomy and histopathologic exam confirmed xanthogranulomatous cholecystitis

Xanthogranulomatous cholecystitis (XGC) is a rare variant of chronic cholecystitis. Despite the fact that it is a benign disease, the inflammatory process may be infiltrative and locally destructive. There is asymmetrical thickening of the gallbladder wall, with the inflammatory process extending into liver parenchyma, omentum, and duodenum (Fig. 24) [62]. Because of close resemblance of the imaging findings, patients with XGC might undergo radical surgical procedures with a misdiagnosis of primary gallbladder adenocarcinoma. The adjacent liver parenchyma may also show abnormal enhancement which may falsely suggest parenchymal invasion. Enlarged lymph nodes may be seen in both gallbladder cancer and XGC, and cholelithiasis is common in both. Intramural hypoattenuating nodules on CT in the thickened gallbladder wall may serve as a helpful finding for diagnosing XGC over gallbladder adenocarcinoma. On MRI, the detection of the fatty content in these nodules could be detected by chemical-shift MR imaging [63]. Cholecystectomy may be necessary for definitive diagnosis in certain patients [62].

### Tuberculous cholangitis

**Fig. 25:**
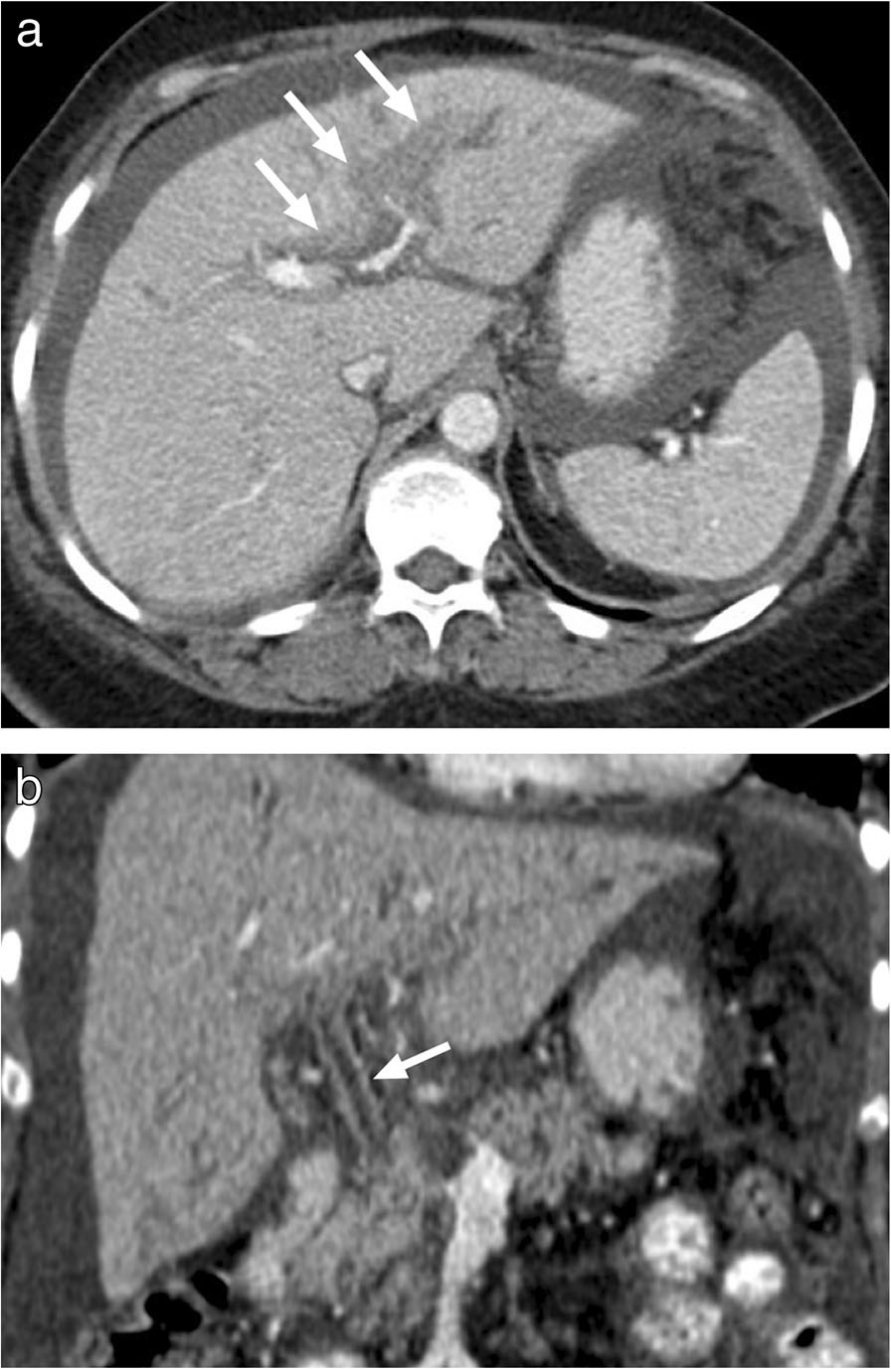
A 68-year-old female with known remote history of breast cancer. The patient recently started to experience abdominal pain, distention, night sweets and weight loss. **a** Axial plane postcontrast CT demonstrated prominent periportal thickening (arrows) in the left liver lobe which may also be seen in infiltrative liver malignancies such as lymphoma. **b** Coronal reformatted image shows significant contrast enhancement in the common bile duct (arrow). Also note moderate ascites with mild peritoneal contrast enhancement which may also suggest peritoneal carcinomatosis. Intraabdominal fluid sampling and cytology confirmed biliary TB

Clinical presentation of biliary involvement in patients with tuberculous cholangitis is usually insidious. Among the commonly reported symptoms are abdominal pain, fever, weight loss, and jaundice [64]. Imaging findings may mimic neoplastic disorders especially in patients with significant mural contrast enhancement and sclerosing biliary obstruction (Fig. 25). Endoscopic retrograde cholangiopancreatography (ERCP) findings may mimic primary sclerosing cholangitis with alternating stenotic and dilated biliary radicals and associated wall irregularities within bile duct walls. These ERCP findings may be seen in other conditions such as leukemia, lymphoma, solid tumor metastases, and hepatic amyloidosis [65].

### IgG4-related sclerosing cholangitis

**Fig. 26:**
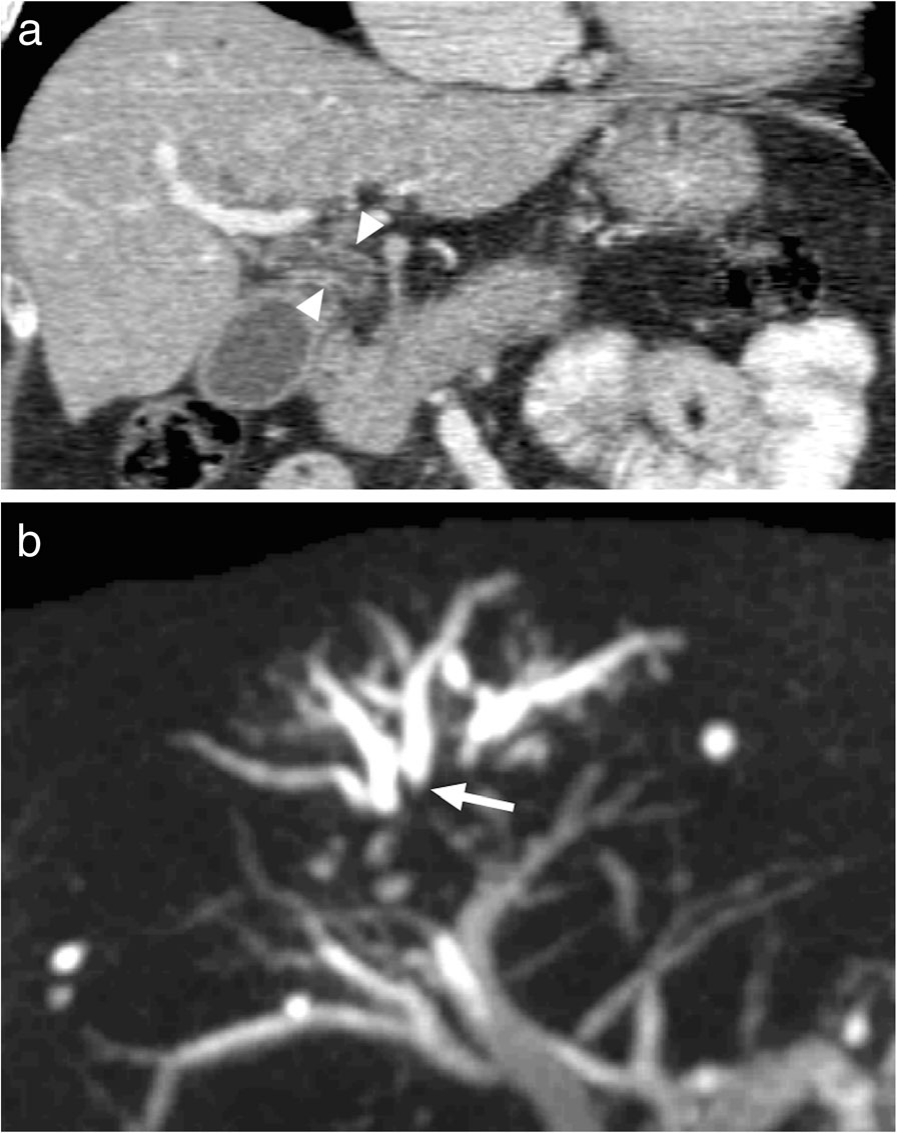
A 43-year-old male presented with jaundice and right upper quadrant pain. **a** Coronally reformatted postcontrast CT image demonstrates diffuse wall thickening in the common bile duct (arrowheads). **b** Coronal plane MIP image from the MRCP study demonstrated abrupt segmental obstruction (arrow) within the same segment. Also note dilated proximal intrahepatic bile ducts. Brush biopsies obtained from endoscopic sampling were suggestive for IgG4-related sclerosing cholangitis and no malignant cells were detected within the specimens. Symptoms improved after appropriate medical treatment

IgG4-related sclerosing cholangitis is frequently associated with autoimmune pancreatitis (AIP). Bile ducts are the most commonly involved body part aside from the pancreas in IgG4-related disease. However, isolated biliary involvement may also be seen without obvious pancreatic involvement [66, 67]. Histopathologically, biliary involvement is characterized with infiltration of bile ducts with IgG4-positive plasma cells and associated extensive fibrosis. Both intrahepatic and extrahepatic bile ducts may be involved in IgG4-related sclerosing cholangitis in diffuse or isolated patterns. Intrapancreatic distal portion of the common bile duct is the most commonly affected biliary segment [66, 68]. Wall thickening and irregularity with associated contrast enhancement and luminal stenosis are typical imaging findings. Focal involvement of distal common bile duct or hilar hepatic involvement with upstream dilation of biliary system may closely mimic a hepatic cholangiocarcinoma or a pancreatic head mass (Fig. 26) [66, 68].

On CT, biliary involvement is typically seen as focal or diffuse wall thickening with contrast enhancement. Biliary stricture with associated enhancing soft tissue mass may also be seen on postcontrast studies which can simulate a cholangiocarcinoma [66, 68].

In IgG4-related sclerosing cholangitis, CT and MRI typically demonstrates concentric soft-tissue thickening of the bile duct wall with homogeneous enhancement in the delayed phase. Associated luminal irregularities are found not only in stenotic segments or in the vicinity of the soft tissue mass but also in areas without any sign of stenosis [68]. Although a thick, annular rind of soft-tissue encasing the affected duct suggests IgG4-related sclerosing cholangitis, this finding may also be seen, to a milder degree, in patients with primary sclerosing cholangitis or infectious cholangitis [66]. Although elevated serum IgG4 levels and associated extrabiliary disease (especially the involvement of the pancreas or the kidneys) may act as a supportive finding, in some patients percutaneous or endoscopic biopsy may be necessary.

## Pancreas

Pancreatic malignancies have significant morbidity and mortality. Pancreatic adenocarcinoma has dismal survival rates which have not changed significantly over the last few decades, despite advances in medical and surgical oncology. Around 90% of patients who were diagnosed with pancreatic cancer are expected to die eventually from the disease [69]. Acute or chronic pancreatitis are common pancreatic diseases that may sometimes be confused with pancreatic cancers.

### IgG4-related autoimmune pancreatitis

AIP is the most common manifestation of IgG4-related systemic disease. AIP type 1 (also known as lymphoplasmacytic sclerosing pancreatitis) is seen mostly in middle aged men, whereas AIP type 2 is frequently a disease of a younger population [67]. Type 1 AIP represents the pancreatic manifestation of systemic IgG4-related disease and type 2 AIP is typically a pancreas-specific disorder with no extrapancreatic involvement (e.g., proximal biliary stricture, submandibular gland involvement, or retroperitoneal fibrosis) [70]. Imaging-based differentiation between these two subtypes is currently not possible and differentiation is based mainly on histopathological evaluation [71].

**Fig. 27:**
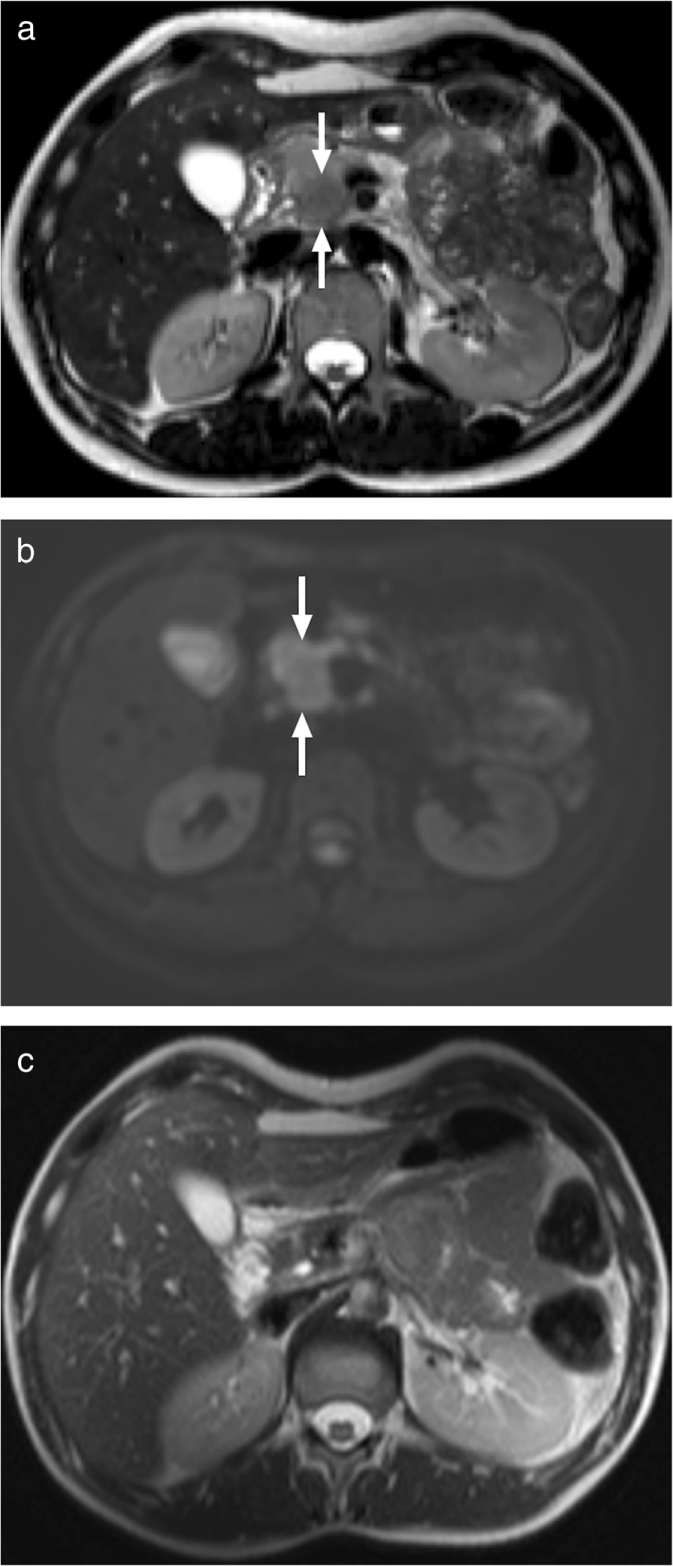
A 32-year-old female with no known medical history presented with chronic epigastric pain and weight loss. Abdominal CT showed a questionable and poorly discernible hypoattenuating mass within the pancreatic uncinate process (not shown). **a** Axial plane T2W MRI clearly demonstrates a hypointense lesion (arrows) in the uncinate process. **b** Axial plane DWI image shows focal diffusion restriction at the lesion site (arrows). The restricted area is larger than the actual lesion which may be a helpful finding for differentiation from adenocancer. Biopsy with endoscopic US guidance and subsequent immunohistochemical staining revealed IgG4-related pancreatic disease. With this result, the patient was placed on steroid treatment. **c** Follow-up imaging 3 months later after systemic steroid treatment confirmed complete resolution of the pancreatic pseudomass

From an imaging standpoint, diffuse enlargement of the pancreas is a commonly encountered finding but focal or mass-like enlargement is not unusual. Diffuse enlargement was reported in between 11 and 56% of patients, whereas focal or mass-like enlargement was observed in 28–59% of the cases (Fig. 27) [72]. Irregular narrowing of the main pancreatic duct is another important feature [67]. Peripancreatic fat planes are typically preserved with no evidence of fluid collection or peripancreatic inflammation, which are commonly seen in acute pancreatitis.

Given the overlap of clinical and imaging features between focal AIP and pancreatic cancer, several studies have sought to differentiate AIP from pancreatic malignancies. Dilation of the main pancreatic duct is mild in patients with AIP as compared to pancreatic adenocarcinoma. Indeed, upstream dilatation of the main pancreatic duct is rarely greater than 4 mm in patients with AIP [73]. The pancreatic duct penetrating through the pseudomass, also known as the duct-penetrating sign, is considered to be a helpful sign for differentiating AIP from pancreatic cancer [67].

### Focal pancreatitis mimicking pancreatic neoplasm

Mass forming focal pancreatitis, by definition, refers to a focal inflammatory process which can sometimes closely mimic pancreatic cancer. Imaging differentiation between a cancer and mass forming focal pancreatitis can sometimes be extremely difficult or impossible [74].

**Fig. 28:**
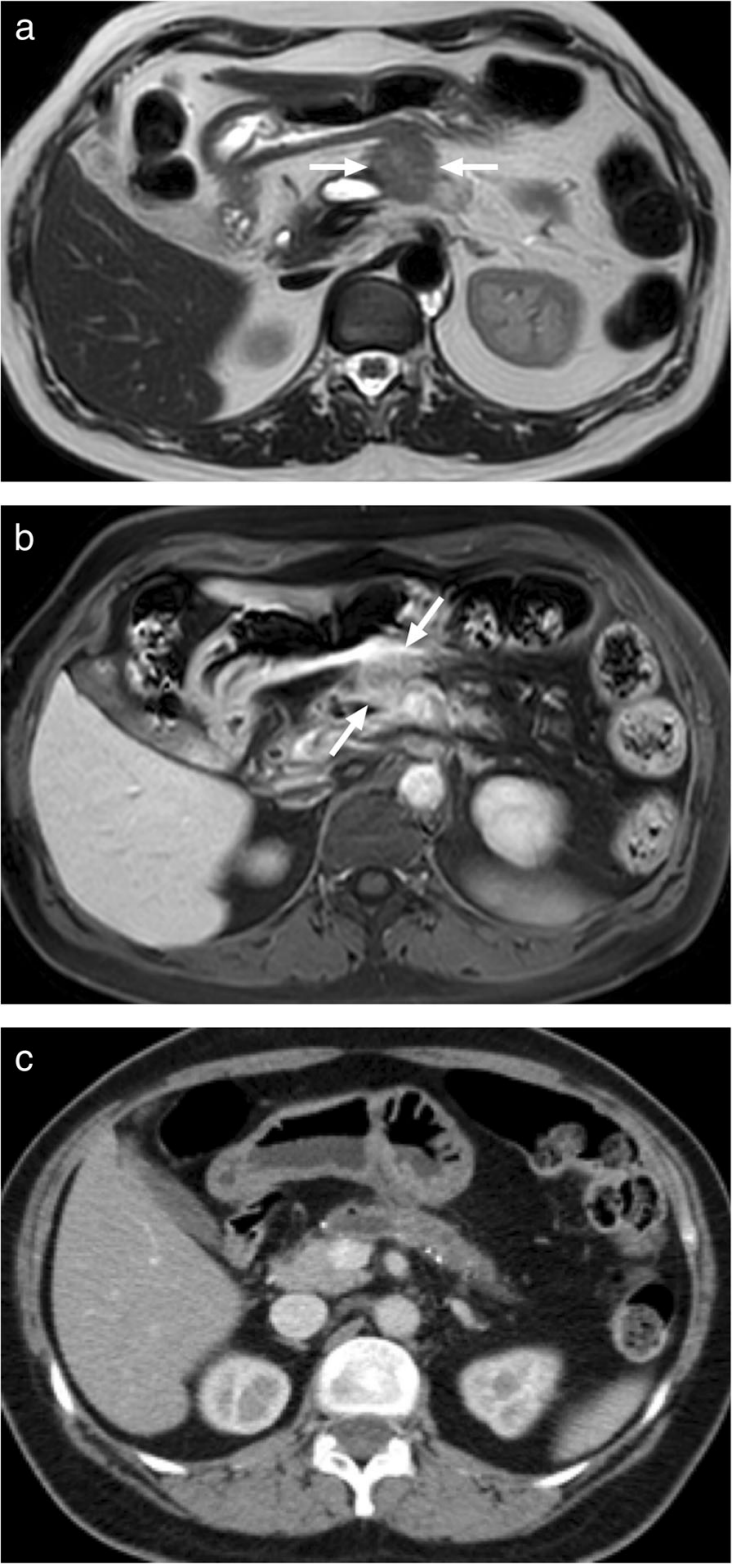
A 54-year-old female with known alcohol induced chronic pancreatitis and chronic pain now presenting with unusually intense epigastric pain. **a** T2W MRI demonstrates a hypointense ill-defined 3-cm-diameter solid mass (arrows) in the pancreatic body/tail with downstream ductal dilatation, likely related to patient’s known chronic pancreatitis. **b** Axial plane postcontrast T1W image demonstrates heterogeneous enhancement of this lesion (arrows). The patient was scheduled for surgical excision. **c** Two weeks after the initial MRI scan, immediately before the planned surgery, the CT scan showed that the mass had almost completely resolved without any treatment. Based on these findings, the mass was considered to represent a pseudomass due to focal pancreatitis

Several studies based on either CT or MRI found no meaningful difference between these two entities in signal characteristics or contrast enhancement patterns [75]. Both are hypo- or isoattenuating to pancreatic parenchyma on CT and are hypointense or isointense on T1-weighted MRI images (Fig. 28). Gradual progressive enhancement is common after contrast injection. The differentiation from a primary adenocarcinoma may be particularly difficult when the clinical presentation does not suggest acute pancreatitis. The abundance of fibrosis in both conditions is probably the underlying reason for these similar imaging features [76]. Histopathologic evaluation or close follow-up imaging is almost always indicated for diagnosis.

### Pancreatic tuberculosis

**Fig. 29:**
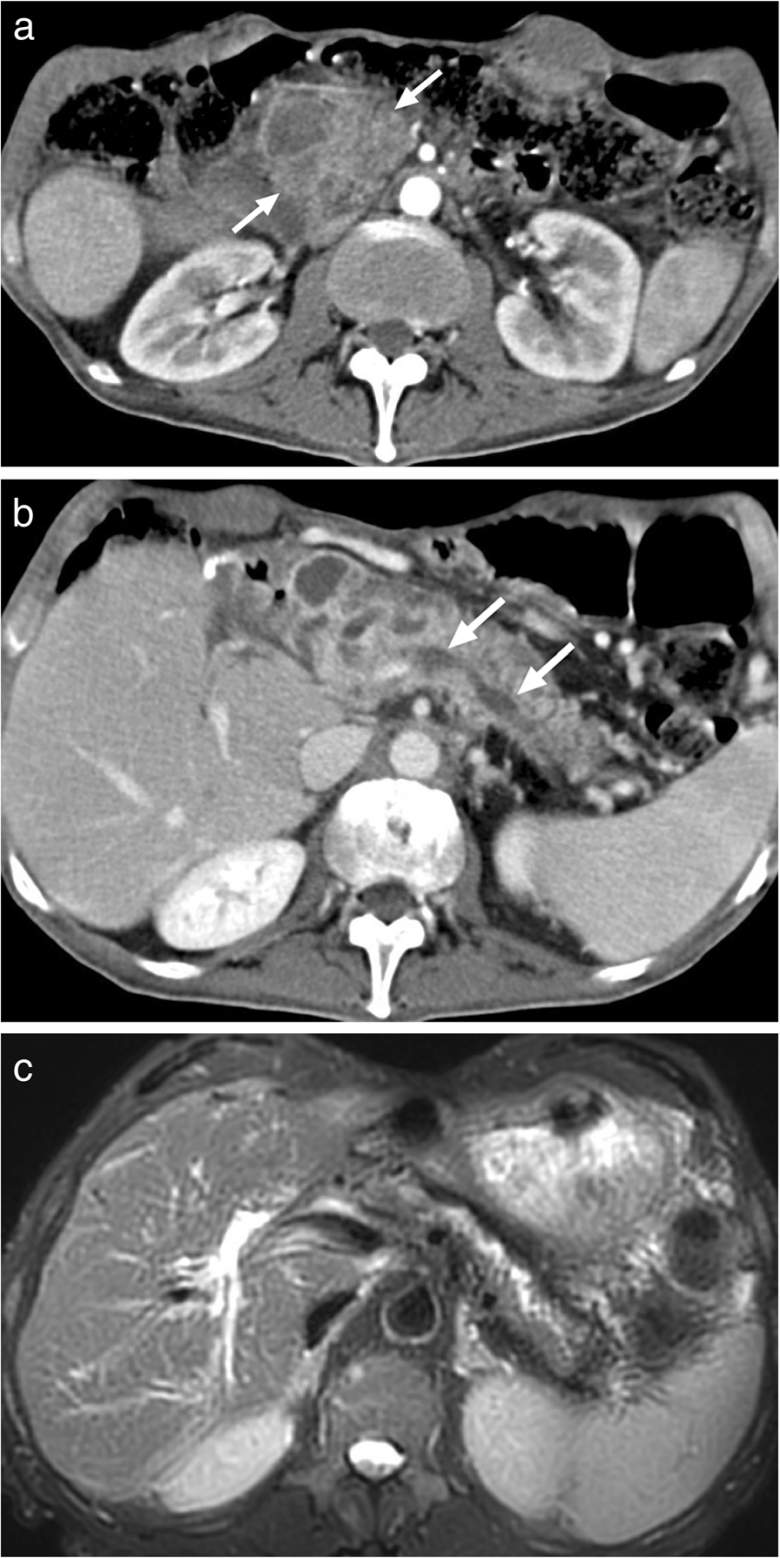
A 62-year-old male with no prior significant medical history presented with gradually increasing epigastric pain and weight loss. **a** Axial plane postcontrast late arterial phase CT image demonstrates diffusely thickened and heterogeneously enhancing pancreas (arrows) with ductal dilatation. **b** There was abrupt cessation of the pancreatic ductal dilatation (arrows) at the location of the pancreatic head mass. Image-guided percutaneous biopsy of the head lesion demonstrated TBC bacillus. The patient was then placed on multidrug antituberculosis treatment. Head, neck, and chest CT scans did not reveal any evidence of disease elsewhere. **c** Axial plane fat-suppressed T2W image 28 months after the initial scan showed significant regression of the pancreatic findings. At the time of this MRI scan, the patient was asymptomatic

Isolated pancreatic tuberculosis (TB) is an extremely rare clinical entity, even in parts of the world where TB is prevalent. Clinical and imaging findings may closely mimic pancreatic cancer and differential diagnosis may be difficult [77]. Abdominal pain, jaundice, and weight loss are the most common presenting symptoms in both clinical situations [78]. The disease may focally or diffusely involve the pancreas. Focal involvement typically presents as a hypoattenuating mass on CT (Fig. 29) and histopathologic examination is almost always necessary for diagnosis. Enlarged peripancreatic lymph nodes with central necrosis may be seen as an associating finding in some patients [79]. Diffuse involvement, a rare presentation of pancreatic adenocarcinoma, may also be difficult to differentiate from primary pancreatic malignancy.

### Pancreatic septic embolus

Septic embolism (SE) is a potentially mortal cause of infection. It can appear in both the early and late phases of infectious diseases. Septic emboli to the brain and the lung have been most extensively studied. Embolic infarcts within pancreatic parenchyma have been reported in patients with multi-organ embolization where brain, spleen and myocardium are also affected [80]. Abdominal pain is a common symptom with associated leukocytosis and elevated amylase levels [81, 82].

**Fig. 30:**
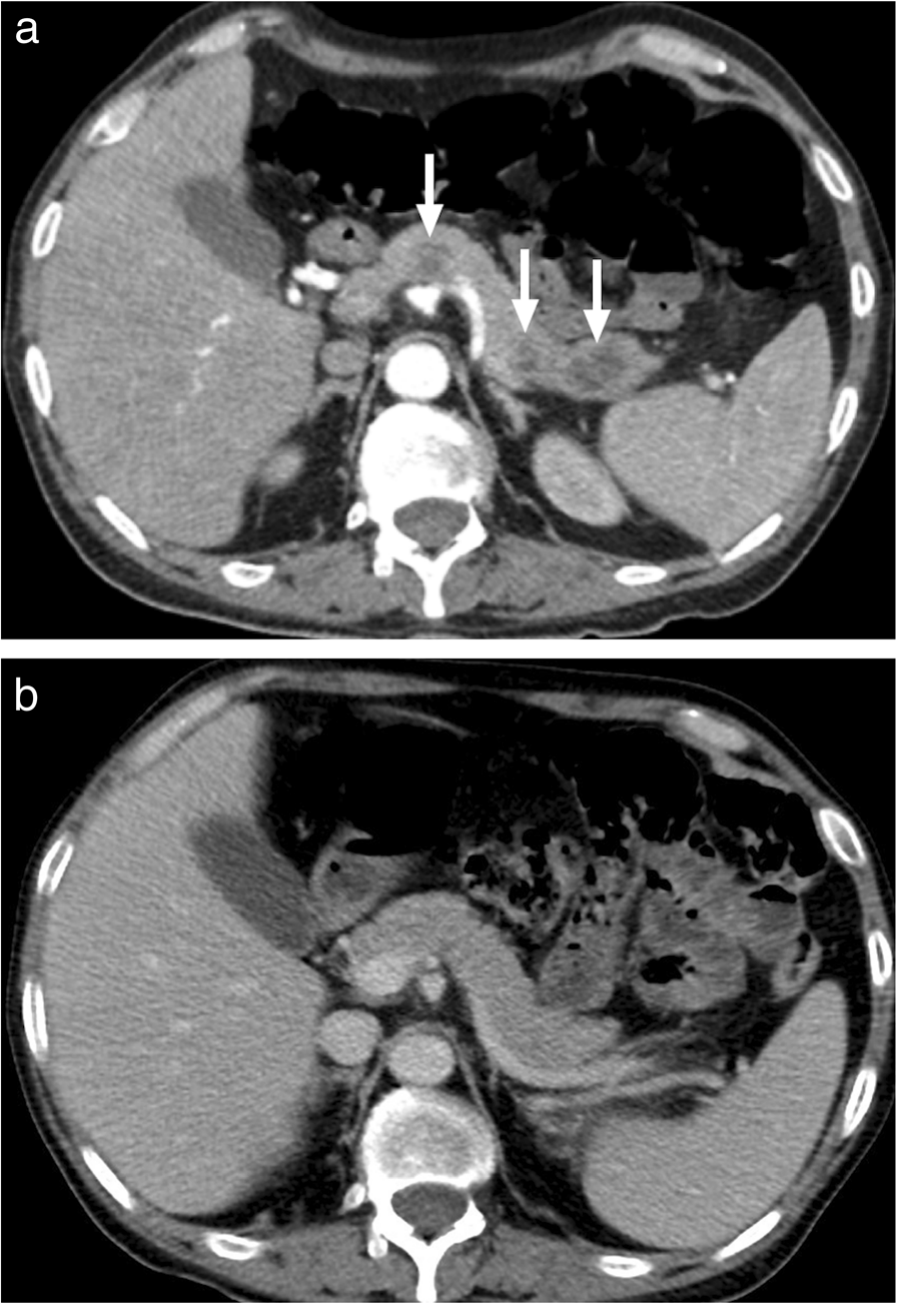
A 65-year-old male with newly diagnosed aggressive lymphoma, undergoing intense chemotherapy in intensive care unit, recently started to complain of fever and epigastric pain. Blood tests were positive for leukocytosis and mildly elevated amylase. **a** Axial plane postcontrast abdominal CT image demonstrates several ill-defined, hypodense lesions within the pancreatic parenchyma (arrows). There was no pancreatic duct dilation or any peripancreatic inflammation/collection. Chest CT study showed innumerable foci of septic emboli within the lung parenchyma. Pancreatic septic emboli or lymphomatous involvement of the pancreas were considered in differential diagnosis. **b** Follow-up abdominal CT 7 days after IV antibiotic treatment, surprisingly demonstrated almost complete disappearance of the focal pancreatic lesions

There is limited information in the literature regarding the imaging findings of pancreatic SE. The embolic foci appeared as hypoattenuating focal lesion within the pancreatic parenchyma, thus mimicking a neoplastic process (Fig. 30). Multifocal hypodense foci may also mimic lymphomatous infiltration or metastases to the pancreas. Despite the fact that multifocal pancreatic adenocarcinoma is rare, that diagnosis should also be considered in the differential diagnosis. Close imaging follow-up after antibiotic treatment is advisable both for confirming diagnosis and for evaluating the response to treatment. Involvement of different organs due to septic emboli, in addition to pancreas, may be a helpful finding for correct differential diagnosis.

## Conclusion

Several non-neoplastic abnormalities may simulate malignant disease and differential diagnosis may be difficult by imaging alone. However, the imaging characteristics and enhancement pattern of the lesion with the assessment of vascular involvement may be very helpful. Co-involvement of the spleen should raise the possibility of an infectious or inflammatory process, as should discrete cystic foci in a solid lesion. Multimodality imaging in relevant cases may also be extremely useful by delineating the different imaging aspects of the same lesion. Besides all these parameters, patient demographics, clinical symptoms, and signs such as fever, laboratory data, tumor markers, short-term changes of imaging findings, and imaging findings of diffusion-weighted imaging (DWI) should all be taken into account for correct diagnosis. Close communication with the referring physician is another important point to be considered. It should be borne in mind that many unusual infectious and non-infectious inflammatory lesions may be sampled for diagnosis with imaging guidance and that this approach is safe in experienced hands and should be liberally used as a tool for early diagnosis.

## Data Availability

Data sharing is not applicable to this article as no datasets were generated or analyzed during the current study.

## Abbreviations

ADC: Apparent diffusion coefficient
AIP: Autoimmune pancreatitis
AML: Acute myeloid leukemia
CCC: Cholangiocellular carcinoma
CT: Computed tomography
DS: Dropped gallstones
DWI: Diffusion-weighted imaging
ELA: Eosinophilic liver abscess
ERCP: Endoscopic retrograde cholangiopancreatography
HCC: Hepatocellular carcinoma
HIV: Human immunodeficiency virus
MRI: Magnetic resonance imaging
PET-CT: Positron emission tomography-computed tomography
SE: Septic embolism
T1W: T1-weighted
T2W: T2-weighted
TB: Tuberculosis
US: Ultrasound
XGC: Xanthogranulomatous cholecystitis

## References

[1] Alobaidi M, Shirkhoda A (2004). Benign focal liver lesions: discrimination from malignant mimickers. Curr Probl Diagn Radiol.

[2] Gandhi Namita S., Feldman Myra K., Le Ott, Morris-Stiff Gareth (2017). Imaging mimics of pancreatic ductal adenocarcinoma. Abdominal Radiology.

[3] Mortele KJ, Segatto E, Ros PR (2004). The infected liver: radiologic-pathologic correlation. Radiographics.

[4] Pastakia B, Shawker T H, Thaler M, O'Leary T, Pizzo P A (1988). Hepatosplenic candidiasis: wheels within wheels. Radiology.

[5] Cornely OA, Bangard C, Jaspers NI (2015). Hepatosplenic candidiasis. Clin Liver Dis (Hoboken).

[6] Metser Ur, Haider Masoom A., Dill-Macky Marcus, Atri Mostafa, Lockwood Gina, Minden Mark (2005). Fungal Liver Infection in Immunocompromised Patients: Depiction with Multiphasic Contrast-enhanced Helical CT. Radiology.

[7] Berlow ME, Spirt BA, Weil L (1984). CT follow-up of hepatic and splenic fungal microabscesses. J Comput Assist Tomogr.

[8] Anttila Veli-Jukka, Lamminen Antti E., Bondestam Sören, Korhola Ossi, Färkkilä Martti, Sivonen Aulikki, Ruutu Tapani, Ruutu Petri (2009). Magnetic resonance imaging is superior to computed tomography and ultrasonography in imaging infectious liver foci in acute leukaemia. European Journal of Haematology.

[9] Semelka R C, Kelekis N L, Sallah S, Worawattanakul S, Ascher S M (1997). Hepatosplenic fungal disease: diagnostic accuracy and spectrum of appearances on MR imaging. American Journal of Roentgenology.

[10] Semelka Richard C., Patrick Shoenut J., Greenberg Howard M., Bow Eric J. (1992). Detection of acute and treated lesions of hepatosplenic candidiasis: Conparison of dynamic contrast-enhanced CT and MR Imaging. Journal of Magnetic Resonance Imaging.

[11] Shirkhoda A, Lopez-Berestein G, Holbert J M, Luna M A (1986). Hepatosplenic fungal infection: CT and pathologic evaluation after treatment with liposomal amphotericin B. Radiology.

[12] Balci N.Cem, Tunacı Atadan, Akınci Ahmet, Cevikbaş Uğur (2001). Granulomatous hepatitis: MRI findings. Magnetic Resonance Imaging.

[13] Karaosmanoglu Ali Devrim, Onur Mehmet Ruhi, Sahani Dushyant V., Tabari Azadeh, Karcaaltincaba Musturay (2016). Hepatobiliary Tuberculosis: Imaging Findings. American Journal of Roentgenology.

[14] Kakkar Chandan, Polnaya Ashwin M., Koteshwara Prakashini, Smiti S., Rajagopal K. V., Arora Ankur (2015). Hepatic tuberculosis: a multimodality imaging review. Insights into Imaging.

[15] ALVAREZ SOL Z (1998). Hepatobiliary tuberculosis. Journal of Gastroenterology and Hepatology.

[16] Jain Rajeev, Sawhney Sukhpal, Gupta Radhika Grover, Acharya Subrat Kumar (1999). Sonographic appearances and percutaneous management of primary tuberculous liver abscess. Journal of Clinical Ultrasound.

[17] Mills P., Saverymuttu S., Fallowfield M., Nussey S., Joseph A.E.A. (1990). Ultrasound in the diagnosis of granulomatous liver disease. Clinical Radiology.

[18] Hickey N., McNulty J. G., Osborne H., Finucane J. (1999). Acute hepatobiliary tuberculosis: a report of two cases and a review of the literature. European Radiology.

[19] Kantarci Mecit, Bayraktutan Ummugulsum, Karabulut Nevzat, Aydinli Bulent, Ogul Hayri, Yuce Ihsan, Calik Muhammet, Eren Suat, Atamanalp Sabri Selcuk, Oto Aytekin (2012). Alveolar Echinococcosis: Spectrum of Findings at Cross-sectional Imaging. RadioGraphics.

[20] Akhan O, Ozmen M N, Dinçer A, Sayek I, Göçmen A (1996). Liver hydatid disease: long-term results of percutaneous treatment. Radiology.

[21] Mortele KJ, Ros PR (2001). Cystic focal liver lesions in the adult: differential CT and MR imaging features. Radiographics.

[22] Land MA, Moinuddin M, Bisno AL (1985). Pyogenic liver abscess: changing epidemiology and prognosis. South Med J.

[23] Ralls PW (1998). Focal inflammatory disease of the liver. Radiol Clin North Am.

[24] Bächler Pablo, Baladron María José, Menias Christine, Beddings Ignacio, Loch Ron, Zalaquett Eugenio, Vargas Matías, Connolly Sarah, Bhalla Sanjeev, Huete Álvaro (2016). Multimodality Imaging of Liver Infections: Differential Diagnosis and Potential Pitfalls. RadioGraphics.

[25] Ralls P W, Colletti P M, Quinn M F, Halls J (1982). Sonographic findings in hepatic amebic abscess. Radiology.

[26] Radin DR, Ralls PW, Colletti PM, Halls JM (1988). CT of amebic liver abscess. American Journal of Roentgenology.

[27] Kwon Jae-Woo, Kim Tae-Wan, Kim Kyung-Mook, Lee So-Hee, Cho Sang-Heon, Min Kyung-Up, Kim You-Young, Park Heung-Woo (2011). Clinical features of clinically diagnosed eosinophilic liver abscesses. Hepatology International.

[28] Vercruysse V, Van Hoe L (2017). Eosinophilic liver disease mimicking hepatic metastases. J Belg Soc Radiol.

[29] Yoo S. Y., Han J. K., Kim Y. H., Kim T. K., Choi B. I., Han M. C. (2003). Focal eosinophilic infiltration in the liver: radiologic findings and clinical course. Abdominal Imaging.

[30] Lim JH, Mairiang E, Ahn GH (2008). Biliary parasitic diseases including clonorchiasis, opisthorchiasis and fascioliasis. Abdom Imaging.

[31] Kaya M, Beştaş R, Cetin S (2011). Clinical presentation and management of Fasciola hepatica infection: single-center experience. World J Gastroenterol.

[32] Talwani R, Gilliam BL, Howell C (2011). Infectious diseases and the liver. Clin Liver Dis.

[33] Maincent G, Labadie H, Fabre M et al (1997) Tertiary hepatic syphilis. A treatable cause of multinodular liver. Dig Dis Sci 42(2):447–450 10.1023/a:1018855011180 9052534

[34] Veeravahu M (1985). Diagnosis of liver involvement in early syphilis. A critical review. Arch Intern Med.

[35] Shim HJ (2010). Tertiary syphilis mimicking hepatic metastases of underlying primary peritoneal serous carcinoma. World J Hepatol.

[36] Peeters L, Van WV, der Perre Van C, Lagrange W, Verbeke M (2005) Tertiary syphilis presenting as hepatic bull's eye lesions. Acta Gastroenterol Belg 68(4):435–439 16432997

[37] Shapiro MP, Gale ME (1987). Tertiary syphilis of the liver: CT appearance. J Comput Assist Tomogr.

[38] Zaidi SA, Singer C (2002). Gastrointestinal and hepatic manifestations of tickborne diseases in the United States. Clin Infect Dis.

[39] Ortego Terryl J., Hutchins Laura F., Rice Jim, Davis Glenn R. (1986). Tularemic hepatitis presenting as obstructive jaundice. Gastroenterology.

[40] Baptista Mariana Andrade, Swei Lo Denise, Hein Noely, Hirose Maki, Yoshioka Cristina Ryoka Miyao, Ragazzi Selma Lopes Betta, Gilio Alfredo Elias, Ferronato Angela Esposito (2014). Cat-scratch disease presenting as multiple hepatic lesions: case report and literature review. Autopsy and Case Reports.

[41] Stuart SM, Nowicki MJ (1998). Radiological case of the month. Cat-scratch disease with hepatic and splenic involvement. Arch Pediatr Adolesc Med.

[42] Lall T, Shehab TM, Valenstein P (2010). Isolated hepatic actinomycosis: a case report. J Med Case Rep.

[43] Islam Tasbirul, Athar Muhammad Nauman, Athar Muhammad Kamran, Usman Mohammed Haris Umer, Misbah Baqir (2005). Hepatic Actinomycosis with Infiltration of the Diaphragm and Right Lung: A Case Report. Canadian Respiratory Journal.

[44] Kim Hee Seoung, Park Noh Hyun, Park Keoung Ah, Kang Soon Beom (2007). A Case of Pelvic Actinomycosis with Hepatic Actinomycotic Pseudotumor. Gynecologic and Obstetric Investigation.

[45] Koyama Takashi, Ueda Hiroyuki, Togashi Kaori, Umeoka Shigeaki, Kataoka Masako, Nagai Sonoko (2004). Radiologic Manifestations of Sarcoidosis in Various Organs. RadioGraphics.

[46] Warshauer DM, Lee JK (2004). Imaging manifestations of abdominal sarcoidosis. AJR Am J Roentgenol.

[47] Dourakis Spyridon P., Cokkinos Demosthenis D., Soultati Aspasia S., Alexopoulou Alexandra, Nezi Vasiliki, Archimandritis Athanasios J. (2007). A case of liver sarcoidosis mimicking cirrhosis. Clinical Imaging.

[48] Deshpande V, Zen Y, Chan JK et al (2012) Consensus statement on the pathology of IgG4-related disease. Mod Pathol 25(9):1181–1192 10.1038/modpathol.2012.72 22596100

[49] Chen JH, Deshpande V (2017). IgG4-related disease and the liver. Gastroenterol Clin North Am.

[50] Zen Yoh, Fujii Takahiko, Sato Yasunori, Masuda Shinji, Nakanuma Yasuni (2007). Pathological classification of hepatic inflammatory pseudotumor with respect to IgG4-related disease. Modern Pathology.

[51] Akatsu Tomotaka, Wakabayashi Go, Tanimoto Akihiro, Kameyama Kaori, Kitajima Masaki (2006). Inflammatory Pseudotumor of the Liver Masquerading as Hepatocellular Carcinoma After a Hepatitis B Virus Infection: Report of a Case. Surgery Today.

[52] Pack GT, Baker HW (1953). Total right hepatic lobectomy; report of a case. Ann Surg.

[53] Honmyo Naruhiko, Kobayashi Tsuyoshi, Tashiro Hirotaka, Ishiyama Kohei, Ide Kentaro, Tahara Hiroyuki, Ohira Masahiro, Kuroda Shintaro, Arihiro Koji, Ohdan Hideki (2016). Inflammatory pseudotumor of the liver occurring during the course of hepatitis C virus-related hepatocellular carcinoma treatment: A case report. International Journal of Surgery Case Reports.

[54] Sakai Masato, Ikeda Hitoshi, Suzuki Norio, Takahashi Atsushi, Kuroiwa Minoru, Hirato Junko, Hatakeyama Shin-itsu, Tsuchida Yoshiaki (2001). Inflammatory pseudotumor of the liver: Case report and review of the literature. Journal of Pediatric Surgery.

[55] Chen Chia-Bang, Chou Chen-Te, Hsueh Ching, Lee Kwo-Whei, Chen Yao-Li (2013). Hepatic inflammatory pseudotumor mimicking hepatocellular carcinoma. Journal of the Chinese Medical Association.

[56] Caramella T, Novellas S, Fournol M, Saint-Paul MC, Bruneton JN, Chevallier P (2007) Imaging of inflammatory pseudotumors of the liver. J Radiol 88(6):882–888 10.1016/s0221-0363(07)89890-8 17652982

[57] Roux Marion, Baranes Laurence, Decaens Thomas, Cherqui Daniel, Nhieu Jeanne Tran Van, Pigneur Frederic, Djabbari Marjan, Levy Mickael, Laurent Alexis, Rahmouni Alain, Luciani Alain (2013). Recurring multicystic inflammatory pseudotumor of the liver: A case report. Clinics and Research in Hepatology and Gastroenterology.

[58] Ramamurthy Nitin K., Rudralingam Velauthan, Martin Derrick F., Galloway Simon W., Sukumar Sathi A. (2013). Out of Sight but Kept in Mind: Complications and Imitations of Dropped Gallstones. American Journal of Roentgenology.

[59] Sathesh-Kumar T (2004). Spilled gall stones during laparoscopic cholecystectomy: a review of the literature. Postgraduate Medical Journal.

[60] Rice David C., Memon Muhammed A., Jamison Richard L., Agnessi Tischa, Ilstrup Duane, Bannon Michael B., Farnell Michael B., Grant Clive S., Sarr Michael G., Thompson Geoffrey B., van Heerden Jonathan A., Zietlow Scott P., Donohue John H. (1997). Long-term consequences of intraoperative spillage of bile and gallstones during laparoscopic cholecystectomy. Journal of Gastrointestinal Surgery.

[61] Levy AD, Murakata LA, Rohrmann CA (2001). Gallbladder carcinoma: radiologic-pathologic correlation. Radiographics.

[62] Ros PR, Goodman ZD (1997). Xanthogranulomatous cholecystitis versus gallbladder carcinoma. Radiology.

[63] Zhao Feng, Lu Pu-Xuan, Yan Sen-Xiang, Wang Gao-Feng, Yuan Jing, Zhang Shi-Zheng, Wang Yi-Xiang J. (2013). CT and MR features of xanthogranulomatous cholecystitis: An analysis of consecutive 49 cases. European Journal of Radiology.

[64] Saluja SS, Ray S, Pal S et al (2007) Hepatobiliary and pancreatic tuberculosis: a two decade experience. BMC Surg 7:10 10.1186/1471-2482-7-10 PMC1925057 17588265

[65] Fan ST, Ng IO, Choi TK, Lai E (1989) Tuberculosis of the bile duct: a rare cause of biliary stricture. Am J Gastroenterol 84(4):413–414 2929563

[66] Vlachou Paraskevi A., Khalili Korosh, Jang Hyun-Jung, Fischer Sandra, Hirschfield Gideon M., Kim Tae Kyoung (2011). IgG4-related Sclerosing Disease: Autoimmune Pancreatitis and Extrapancreatic Manifestations. RadioGraphics.

[67] Hedgire Sandeep S., McDermott Shaunagh, Borczuk David, Elmi Azadeh, Saini Sanjay, Harisinghani Mukesh G. (2013). The Spectrum of IgG4-Related Disease in the Abdomen and Pelvis. American Journal of Roentgenology.

[68] Martínez-de-Alegría Anxo, Baleato-González Sandra, García-Figueiras Roberto, Bermúdez-Naveira Anaberta, Abdulkader-Nallib Ihab, Díaz-Peromingo José A., Villalba-Martín Carmen (2015). IgG4-related Disease from Head to Toe. RadioGraphics.

[69] Ryan DP, Hong TS, Bardeesy N (2014). Pancreatic adenocarcinoma. N Engl J Med.

[70] Hart PA, Zen Y, Chari ST (2015). Recent advances in autoimmune pancreatitis. Gastroenterology.

[71] Zhang Lizhi, Chari Suresh, Smyrk Thomas C., Deshpande Vikram, Klöppel Günter, Kojima Motohiro, Liu Xiuli, Longnecker Daniel S., Mino-Kenudson Mari, Notohara Kenji, Rodriguez-Justo Manuel, Srivastava Amitabh, Zamboni Giuseppe, Zen Yoh (2011). Autoimmune Pancreatitis (AIP) Type 1 and Type 2. Pancreas.

[72] Lee LK, Sahani DV (2014). Autoimmune pancreatitis in the context of IgG4-related disease: review of imaging findings. World J Gastroenterol.

[73] Wakabayashi Tokio, Kawaura Yukimitsu, Satomura Yoshitake, Watanabe Hiroyuki, Motoo Yoshiharu, Okai Takashi, Sawabu Norio (2003). Clinical and imaging features of autoimmune pancreatitis with focal pancreatic swelling or mass formation: comparison with so-called tumor-forming pancreatitis and pancreatic carcinoma. The American Journal of Gastroenterology.

[74] Fattahi Rana, Balci N. Cem, Perman William H., Hsueh Eddy C., Alkaade Samer, Havlioglu Necat, Burton Frank R. (2009). Pancreatic diffusion-weighted imaging (DWI): Comparison between mass-forming focal pancreatitis (FP), pancreatic cancer (PC), and normal pancreas. Journal of Magnetic Resonance Imaging.

[75] Patlas M, Deitel W, Taylor B, Gallinger S, Wilson SR (2007) Focal chronic pancreatitis mimicking pancreatic head carcinoma: are there suggestive features on ultrasound? Can Assoc Radiol J 58(1):15–21 17408158

[76] Johnson PT, Outwater EK (1999). Pancreatic carcinoma versus chronic pancreatitis: dynamic MR imaging. Radiology.

[77] Pramesh C.S., Heroor A.A., Gupta S.G., Krishnamurthy S., Shukla P.J., Jagannath P., DeSouza L.J. (2003). Pancreatic tuberculosis: an elusive diagnosis. HPB.

[78] Zacharia G. S., Antony R., Kolassery S., Ramachandran T. M. (2014). Isolated pancreatic tuberculosis masquerading as pancreatic cancer. Gastroenterology Report.

[79] Falkowski AL, Graber J, Haack HG, Tarr PE, Rasch H (2013) Isolated pancreatic tuberculosis: a case report and radiological comparison with cystic pancreatic lesions. J Radiol Case Rep 7(1):1–11 10.3941/jrcr.v7i1.1292 PMC3557128 23372869

[80] Hart RG, Kagan-Hallet K, Joerns SE (1987). Mechanisms of intracranial hemorrhage in infective endocarditis. Stroke.

[81] Haigh E (1956). Acute pancreatitis in childhood. Arch Dis Child.

[82] Sheil A. G. R., Reeve T. S., Little J. M., Coupland G. A. E., Loewenthal John (1969). Aorto-intestinal fistulas following operations on the abdominal aorta and iliac arteries. British Journal of Surgery.

